# Indirect state-level estimation of sexual minority adolescent populations by sex, age, and race/ethnicity using random forests

**DOI:** 10.1371/journal.pone.0349759

**Published:** 2026-06-09

**Authors:** Bruno Alves-Maciel, Sanjana Pampati, Ari Fodeman, Michelle Van Handel, Lijia Zheng, Ziping Ye, Reza Yaesoubi, Joshua A. Salomon

**Affiliations:** 1 Department of Health Policy and Management, Yale School of Public Health, Yale University, New Haven, United States of America; 2 Division of Adolescent and School Health, National Center for Chronic Disease Prevention and Health Promotion, Centers for Disease Control and Prevention, Atlanta, United States of America; 3 Office of the Director, National Center for HIV, Viral Hepatitis, STD and TB Prevention, Centers for Disease Control and Prevention, Atlanta, United States of America; 4 Department of Health Policy, Stanford School of Medicine, Stanford University, Stanford, United States of America; Northern Arizona University, UNITED STATES OF AMERICA

## Abstract

**Purpose:**

Estimating population sizes of adolescents who identify as lesbian, gay, or bisexual (LGB) is important for addressing health needs and disparities. Most states have Youth Risk Behavior Surveys (YRBS) among high school students, but not all include an item about sexual identity. This study’s aim was to estimate the percentages of students identifying as LGB stratified by sex, age, and race and ethnicity where state data is incomplete. States where 2021 YRBS data are not available are outside the scope of this study.

**Methods:**

We developed two random forests trained separately for each sex and evaluated the models’ performance in predicting percentages of respondents identifying as LGB by state and demographic strata. We then estimated percentages for states that did not include or have responses to the LGB identity question available in 2021.

**Results:**

The random forests outperformed benchmark comparison models based on a simple logistic regression approach. Estimates of students who identify as LGB across demographic strata and states ranged 5%–30%. The estimated percentages for states that did not ask students about sexual identity fell within the same range.

**Conclusion:**

Our approach to deriving state-level estimates of LGB students by sex, race and ethnicity, and age performs well and can be used to inform efforts to improve health and well-being of LGB youth.

## Introduction

Adolescents who identify as lesbian, gay, or bisexual (LGB) face stigma, discrimination, and increased risk for violence victimization, sexually transmitted infections, early pregnancy, and poor mental health outcomes [1–4]. Accurately estimating the percentages of youth who identify as LGB is crucial for health and education agencies aiming to address students’ health and well-being. Development, implementation, and assessment of practices and programs require understanding differences in LGB identification across subgroups—including by sex, age, and race and ethnicity—but data and understanding of subgroup disparities among LGB populations are limited [5]. For example, state or local public health programs focused on addressing HIV risk may benefit from detailed subgroup data so outreach materials can be tailored accordingly.

The Youth Risk Behavioral Surveillance System includes surveys among national and state-level samples of high school students. The Youth Risk Behavior Survey (YRBS) is conducted every 2 years among students enrolled in grades 9–12. In 2021, the high school YRBS was offered in 45 states, of which 35 included a question about sexual identity [6,7]. Respondents predicted to self-identify as lesbian, gay, or bisexual if the sexual identity questions were asked in their state’s YRBS are referred to as S-LGB throughout. Given gaps in reported sexual identity among adolescents in specific states, machine learning models have been developed to estimate the percentages of YRBS S-LGB in states that administer the high school YRBS but do not include the sexual identity question [8]. Random forest (RF) modeling produced robust estimates of the S-LGB percentage in states that did have YRBS data from this item. However, estimates were made at the state level, without further stratification by sex, race and ethnicity, or age. Detailed estimates of LGB youth within specific demographic groups can help local education and health agencies design and evaluate programs, guide service delivery and resource allocation, and inform epidemiological and economic modeling [9,10].

In this paper, we expand upon previous work by developing a systematic framework for estimating S-LGB percentages among high school students in subgroups defined by sex, race and ethnicity, and age. We develop RF models, benchmark their performance against simpler imputation approaches, and create estimates in detailed strata for states that administered the 2021 YRBS but did not have available data on LGB identity. States that did not administer or have any data available for the 2021 YRBS are outside the scope of our study.

## Materials and methods

### Data

We used combined state-level 2021 high school YRBS datasets compiled by Centers for Disease Control and Prevention for public use [7]. The high school YRBS is administered to students in grades 9–12 every other year to collect information about demographics, health behaviors, health conditions, and life experiences. The 2021 standard questionnaire includes this question regarding sexual identity:

*Q. Which of the following best describes you?*

A *Heterosexual (straight)*
B *Gay or lesbian*
C *Bisexual*
D *I describe my sexuality some other way*
E *I am not sure about my sexual identity (questioning)*
F *I do not know what this question is asking*

Respondents were considered to self-report LGB identity if they answered this identity question with options B (gay or lesbian) or C (bisexual). Since our goal was to estimate the percentage of LGB respondents, we coded our response variable as 1 for respondents who marked B or C in this question, and 0 for all other respondents (including those who did not respond on this item). Thus, respondents coded as 0 are not necessarily heterosexual but comprise a heterogeneous group that does not self-identify as LGB.

In 2021, 43 states administered a high school YRBS and made the survey data publicly available through CDC, but eight of these either did not include or did not have data available for the sexual identity question. Those eight states are referred to here as **LGB-N**, whereas states that include the sexual identity question are referred to as **LGB-Y**. We estimated the S-LGB population in the LGB-N states’ overall population, and subgroups stratified by sex, race and ethnicity, and age. These demographic characteristics are collected in each state’s questionnaire in addition to behavioral questions, experiences, height, weight and school grade. Those demographic characteristics of the sample are summarized in Table 1, with details in S1 and S2 Tables.

**Table 1:** Description of study sample, by sex, race and ethnicity, and age.

Characteristics	Options	Respondents when pooling all states N (%)	Median respondents across state samples N (%)
Sex	Female	72,838 (49.2)	833 (49.2)
	Male	73,235 (49.5)	863 (49.4)
	Missing	1830 (1.2)	16 (0.9)
Race and ethnicity	Non-Hispanic White	79,439 (53.7)	791 (50.3)
	Non-Hispanic Black	18,540 (12.5)	147 (9.6)
	Hispanic	26,646 (18.0)	315 (15.6)
	Non-Hispanic American Indian/ Alaska Native, Asian, Native Hawaiian, Other Pacific Islander, or Non-Hispanic Multiracial^*^	19,330 (13.1)	173 (9.6)
Age	14-year-olds and below	32,005 (21.6)	360 (21.1)
	15-year-olds	40,540 (27.4)	477 (27.5)
	16-year-olds	36,015 (24.4)	425 (24.2)
	17-year-olds	30,430 (20.6)	378 (20.5)
	18-year-olds and above	8623 (5.8)	109 (5.8)
	Missing	326 (0.2)	3 (0.2)

* Given the variety of races and ethnicities grouped into the same category, this group is referred to as “Other” in subsequent figures to save space.

Note: Data are from 2021 pooled Youth Risk Behavior Survey state sample. Respondent numbers and percentages reflect unweighted values.

#### Missing responses.

Whereas missing responses to the identity question were considered as non-S-LGB, missingness in other YRBS responses was addressed through two steps: new binary variables for each YRBS question indicate whether the response was missing. Then missing values in the original question were overwritten with the mode across all valid responses for each question. If a question was not asked in any of the eight LGB-N states, it was excluded from analysis. If at least one of the eight states included the question, all responses would be considered missing for states that did not include it. Thus, states that did not ask a question included in the analysis would have no variance in the responses for that question, with all assigned the mode for that item.

### Estimation models by demographic subgroups

A previous study compared eight approaches to estimate the S-LGB percentage in each state [8]. Those approaches predicted whether each respondent in a state identified as S-LGB. For each approach, predictions for each respondent were aggregated into a percentage of S-LGB per state using YRBS sampling weights. Among those eight previous approaches, the RF obtained the best performance. Therefore, in this analysis, we focus on variations of the RF model only.

We define an “estimation approach” based on the scope of input data along the demographic subgroups of sex, race and ethnicity, and age. We consider two categories for sex, four for race/ethnicity (from the YRBS dataset’s “race4“field) and five categories for age, pooling the existing levels into 14 years old and below, 15, 16, 17, and 18 years old and above. We evaluated nine estimation approaches listed below. Each approach used one or more RFs to calculate S-LGB probabilities for each YRBS respondent. The simplest approach (Aggregate) trained a single RF on all available responses. However, an approach segmented by sex (*S*^*x*^) trained two RFs—one on data from female respondents, and another on data from male respondents. As we increased the segmentation of the input data, more RFs were trained in datasets that were smaller and potentially less balanced. Each of those RFs separately estimated S-LGB probabilities for respondents within their demographic subgroup only—meaning a RF trained on data from female respondents estimated the S-LGB probabilities of each female respondent in LGB-N states, but no male respondents. By combining the predictions from all trained RFs, we created a dataset with S-LGB probabilities for all respondents.

Aggregate S-LGB percentages in any demographic subgroup were estimated by pooling individual probabilities based on the respondents’ demographic characteristics, regardless of the estimation approach. The estimation approach refers to how the input data are organized for training RFs, whereas the “prediction subgroup” refers to how the output probabilities are grouped to calculate S-LGB percentages.

#### Validation.

To evaluate and select which estimation approach best predicts the percentage of S-LGB respondents in an LGB-N state, we used leave-one-group-out (LOGO) validation. Each LGB-Y state (states that have data available for the sexual identity question) was one of the LOGO groups. LOGO mimics having multiple states for training data (i.e., LGB-Y states), then uses the trained model to estimate S-LGB probabilities in the left-out state (standing in for an LGB-N state). The process was repeated by leaving out each LGB-Y state once. Once a group of at least 35 models was trained and tested across all LGB-Y states, we used those estimated probabilities to calculate the S-LGB percentage for respondents of each prediction subgroup. Those percentages were used to calculate metrics to compare each estimation approach. This study focuses on aggregated percentages, which provides more leeway for individual classification errors as long as the overall percentage is consistent with the observed values. This study does not aim to predict the response of individual students. The RF estimation approaches in this study are listed below.

Aggregate: No segmentation, training 35 models for LOGO and 1 for LGB-N states
*S*^*x*^: Segmentation by sex, 70 models for LOGO and 2 in LGB-N states
*S*^*e*^: Segmentation by race/ethnicity, 140 models for LOGO and 4 in LGB-N states
*S*^*a*^: Segmentation by age, 175 models for LOGO and 5 for LGB-N states
*S*^*xe*^: Segmentation by sex and race/ethnicity, 280 models for LOGO and 8 for LGB-N states
*S*^*xa*^: Segmentation by sex and age, 350 models for LOGO and 10 for LGB-N states
*S*^*ea*^: Segmentation by race/ethnicity and age, 700 models for LOGO and 20 for LGB-N states
*S*^*xea*^: Segmentation by sex, race/ethnicity, and age, 1400 models for LOGO and 40 for LGB-N states

#### Metrics.

Two metrics were calculated to compare the performance of different predictive models: the Total Squared Error (TSE), and the Intraclass Correlation Coefficient (ICC). They reflect not only the difference between the estimated and real S-LGB percentages, but also how that difference is distributed between states in the validation step (i.e., how closely the variance of estimates matches that of the real percentages). We computed the ICC following the formulation in Shrout and Fleiss (1979) [11–13]: variance between states/(variance of estimation error variance between states+variance of estimation error). Compared to other traditional error metrics that could measure the RF error directly (e.g., cross-entropy), the ICC applied to the aggregated percentages allows us to measure how consistent our predictions are with the observed values for each state (i.e., the observed difference in percentages between states should be preserved by the RF estimator). A high ICC would suggest that not only is the prediction close to the real value, but are also consistently differentiated between the states.

#### Hyperparameter tuning.

Each RF in each estimation approach has its own set of adjustable hyperparameters that configure how the RF will be trained, but they do not directly interact with the input or output data. Due to the number of models that need to be trained to test each configuration, we tuned only the “Aggregate” estimation approach for validation. All other estimation approaches assumed the same RF configuration was acceptable. The hyperparameter tuning was also restricted to a subset of possible values, for only some of the RF hyperparameters (considering the *RandomForestClassifier* in Python [14]). An arbitrary number of 100 random combinations from this subset were tested to limit computational usage. The hyperparameters were chosen through the ICC obtained across states in the LOGO validation to guarantee that the resulting RF would be capable of generating consistent overall percentages for different states, rather than focusing solely on the individual classification of students. S1 Appendix details hyperparameter selection. The selected hyperparameter values were 150 random trees per forest, each with a maximum depth of 14, splitting only when there were more than 15 samples. Each node could consider up to one-third of the total parameters as splitting criteria. Bootstrapping was applied to the training set to promote generalization as follows: each of the 150 random trees was trained on an artificial dataset with the same size as the input training set but created by randomly sampling responses from the training set with repetition.

#### Logistic regression benchmark.

We benchmarked the performance of the RFs against a comparator model that is more straightforward to compute and understand, but still reasonable as an empirically derived alternative. This comparator was a logistic regression that calculated S-LGB probabilities based only on sex, race, and age of each respondent, ignoring all other YRBS questions. Whereas estimates for S-LGB probabilities could be readily obtained with non-specific methods such as using the mean from other states, we benchmark our approach against a logistic regression for a more sophisticated comparison that uses at least some of the available data to provide different estimates for each LGB-N state. The probabilities were aggregated into each prediction subgroup as a comparison against other estimation approaches. The logistic regression benchmark was evaluated through the same LOGO validation (i.e., 35 logistic regression models were trained, one for each of the LGB-Y states).

#### Estimating S-LGB percentages among LGB-N states.

The estimation approach selected through validation was trained on data from all 35 LGB-Y states, then used to predict the S-LGB probabilities for each respondent in LGB-N states. Those probabilities were grouped into the prediction subgroups of interest. Prediction intervals were calculated by assuming the residuals of the estimates in the training set followed a student’s t-distribution and calculating a 95% interval around the mean residual. This prediction interval reflects uncertainty on the model’s ability to appropriately estimate the S-LGB percentages in each LGB-N state. It differs from the confidence interval of the S-LGB percentages in LGB-Y states, which instead shows the 95% interval of estimates from the bootstrapped decision trees of the RF estimator.

## Results

### Model selection through LOGO validation

Table 2 compares the ICC and TSE achieved through different estimation approaches in LOGO validation. All prediction subgroups other than statewide show the median ICC between populations (e.g., the ICC for “each sex” is the median between the ICC of the 35 models trained on male respondents and the ICC of the 35 models trained on female respondents). Table 2 shows only the best-performing RF approach and the logistic regression benchmark. Even between RF approaches, the ICC varied from 0.35 to 0.75. In general, total error increased for estimation approaches using more demographic characteristics, whereas no consistent pattern was seen for ICC.

**Table 2:** Intraclass Correlation Coefficient (ICC) and Total Squared Error (TSE) from the best estimation approach for S-LGB percentage contrasted with the logistic regression.

Prediction Subgroup	Best Approach (ICC; TSE)	Logistic Regression (ICC; TSE)
Statewide	70 RFs on *S*^*x*^ (0.751; 2424)	(−0.211; 2333)
Each sex	70 RFs on *S*^*x*^ (0.733; 2680)	(−0.129; 2710)
Racial groups	70 RFs on *S*^*x*^ (0.611; 2770)	(−0.163; 3232)
Age groups	70 RFs on *S*^*x*^ (0.677; 3350)	(−0.050; 3767)
Sex/race	280 RFs on *S*^*xe*^ (0.607; 4043)	(−0.066; 4053)
Sex/age	70 RFs on *S*^*x*^ (0.615; 4386)	(−0.055; 4826)
Race/age	70 RFs on *S*^*x*^ (0.575; 4827)	(0.119; 5512)
Sex/race/age	70 RFs on *S*^*x*^ (0.466; 6572)	(−0.026; 7492)

Note: The table shows the best estimation approach in each prediction subgroup. The logistic regression had similar Total Squared Error (TSE) to the best Random Forest (RF) approaches, but its Intraclass Correlation Coefficient (ICC) was mostly negative (i.e., incapable of reflecting the variance of the percentage of respondents that self-identify as lesbian, gay, or bisexual in the questionnaire along different states). The group of 70 RF models trained on two separate datasets per state, one for each sex (*S*^*x*^) appeared to have the best balance of ICC and TSE during validation, regardless of the prediction subgroup. A group of 280 RF models trained on eight separate datasets per state, one for each combination of sex and race (*S*^*xe*^), outperforms the 70 RF on *S*^*x*^ only in one case. A complete comparison including all RF approaches is in S2 Appendix, S4 Table.

The RFs trained on separate sexes (*S*^*x*^) achieved consistently high ICC and low TSE compared to other estimation approaches. Even the S-LGB percentages for different race and ethnicity groups or different age groups were better estimated by RFs trained on *S*^*x*^ than on *S*^*e*^ (separate races) or *S*^*a*^ (separate ages). In fact, the estimates from *S*^*x*^ outperformed other estimates in most of the estimation tasks. For subgroups defined by both sex and race, the ICC from the *S*^*x*^-trained RFs had the second-best result, with a difference of 0.04.

The logistic regression produced predictions that fell within a narrow range of the mean S-LGB percentage across all states (around 14%). It did not capture differences between states with percentages farther from the mean. In comparison, the RF estimated a wider range of values (from around 12%–17%). This contrast between LR and RF was similar in all other prediction subgroups.

**Fig 1:**
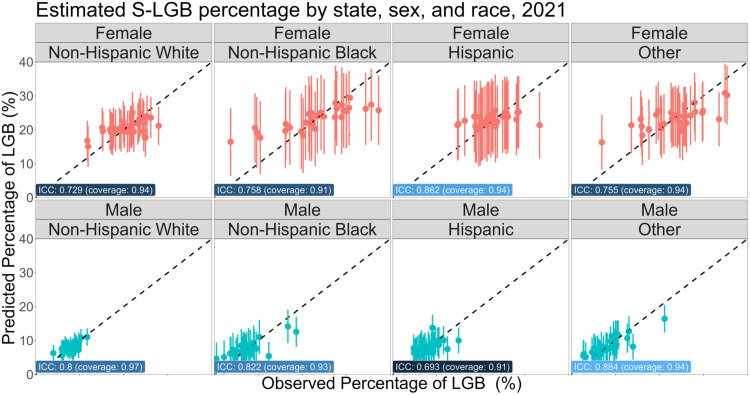
Comparison between the observed and predicted percentage of respondents that self-identify as lesbian, gay, or bisexual in the questionnaire (S-LGB) in each combination of sex, race and ethnicity, and states for which there are data, and the estimates obtained by the random forest models trained on different sexes during the validation process. Note: Each graph lists the Intraclass Correlation Coefficient (ICC) across all states.

The two *S*^*x*^-trained RFs produced clear differences between predictions for male and female students. Fig 1 illustrates a comparison between different racial and ethnic groups and by sex. Considering age instead of race and ethnicity yielded a similar pattern, with modest differences in S-LGB percentages observed along racial and ethnic groups or age groups. S2 Appendix’s Fig S1 to S9 compare other prediction subgroups.

### Application

**Fig 2:**
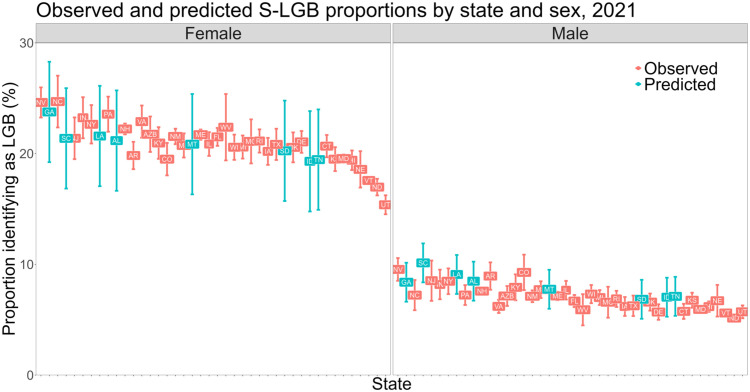
Comparison between the estimates of respondents who self-identify as lesbian, gay, or bisexual in the questionnaire (S-LGB) obtained by the two random forest models trained on different sexes when the prediction subgroup is the sexes.

**Fig 3:**
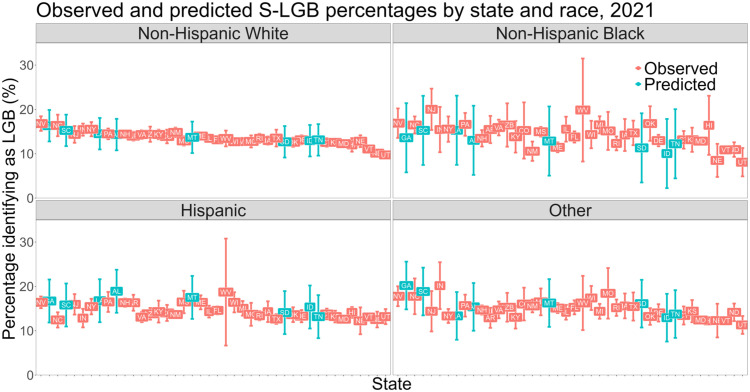
Comparison between the estimates of respondents who self-identify as lesbian, gay, or bisexual in the questionnaire (S-LGB) obtained by the two random forest models trained on different sexes when the prediction subgroup is the race and ethnicity.

For each of the eight LGB-N states, we used the two RF models from the *S*^*x*^ estimation approach to estimate the S-LGB probability for each respondent. We compiled those likelihoods into prediction subgroups (Fig 2). Following CDC analysis conventions, subpopulations with fewer than 30 respondents were omitted. The estimated S-LGB percentages for LGB-N states were spread along the range of observed percentages, rather than landing consistently higher or lower. There are differences between the estimates for males and females, but no systematic differences across racial and ethnic groups or age groups (Fig 3).

Whereas observed S-LGB percentages without stratification varied between 9% (Utah) and 18% (Nevada), the estimated S-LGB percentages in LGB-N states ranged from 13% (Tennessee) to 16% (Georgia). S2 Appendix’s Fig S10 to S16 show other levels of stratification, confirming that the S-LGB percentage in LGB-N states are distributed within the same range as the LGB-Y states.

## Discussion

This study aimed to develop an approach to estimate state-level percentages of high school students identifying as LGB within subgroups defined by sex, age, and race and ethnicity. By estimating the size of the S-LGB population in states that participate in YRBS but do not have data on sexual identity available, public health and education providers can better tailor programs and interventions to best meet the needs of sexual minorities across different demographic characteristics. These findings could also inform epidemiological and economic modeling, which often require estimates of demographic categories as inputs. In the absence of relevant Census and survey data, using data from validated predictor models such as ours is a feasible alternative.

We compared the performance of multiple RF approaches and showed that two RFs trained separately for males and females best estimate S-LGB percentages across demographic subgroups. The clear difference in S-LGB percentages between male and female respondents could be enabling those two RFs to learn differing patterns in the responses of male and female S-LGB students, regardless of age or race and ethnicity. In contrast, no consistent patterns are clear between age or racial and ethnic categories and the RFs trained on those subgroups have limited predictive ability. As a result, even for estimating the S-LGB percentage among students of a specific age or race and ethnicity, those estimates will be more accurate when looking only at patterns between different sexes. Low ICC in substrata of specific demographics could indicate that a specific population does not follow the same pattern as the national population (e.g., the lower ICC for male Hispanic students suggests there could be different response patterns for S-LGB in that subpopulation).

This study’s methods could also train specialized models in different years or YRBS questions—to predict other responses that might be missing in any state. Computational complexity limited the number of options that could be assessed in this study in a timely manner (i.e., several days or weeks of computational time). Training and tuning specialized models for each demographic characteristic found to have a high ICC with the chosen *S*^*x*^ approach would test whether more patterns that led to the high ICC can be captured. Sample size was also a limiting factor, especially for demographics intersections with less than 30 respondents. An underlying assumption is that students from LGB-N states would follow the same patterns captured across LGB-Y states. The high ICC observed in our results suggests that our approach is capable of properly differentiating between states with higher or lower S-LGB population. Thus, it could be that other questions in the survey indirectly capture unmeasured factors or attitudes that affect S-LGB proportions. However, we cannot guarantee that LGB-N states would not be outliers with patterns hitherto unobserved in LGB-Y states. This study proposes an approach to generate better proportion estimates than would be obtained with simpler methods, but no estimates supersede expanding surveillance in those states.

The S-LGB proportions can be used to calculate surveillance indicators among adolescents who self-identify as lesbian, gay, or bisexual to help identify populations at increased risk for morbidity or mortality (e.g., disease or infection rates, chronic disease indicators, etc.). However, such estimates are best used in conjunction with the ICC metric as an indication of the relative predictability of the S-LGB proportions. For population groups where the estimates have low ICC, calculated rates should be interpreted with caution since it could be an indication that no consistent patterns of behavior could be assumed within that population, or at least that none of those patterns are captured by the YRBS questionnaire.

We focused our estimation on those who explicitly identified as lesbian, gay, or bisexual. We could similarly estimate percentages for groups excluded from the S-LGB group through the same method (e.g., students who respond “other” or “questioning” to the sexual identify question). Similarly, future work may consider estimating percentages based on indicators of sexual behavior in addition to sexual identity.

## Conclusion

This study compared eight RF approaches estimating the percentage of YRBS survey respondents who would self-identify as LGB. Those approaches are contrasted with a simpler logistic regression using only sex, race, and age as predictors. All proposed approaches are trained using responses from subsets of data defined by demographic characteristics. In general, RFs perform similarly to a simpler logistic regression benchmark in total absolute error but better reproduce the systematic variation in percentages across states (measured through ICC). The approach validated with highest ICC was a pair of RFs trained separately on respondents of each sex.

Our results suggest the RFs learn a consistent pattern of responses for each sex. Therefore, the two RFs trained on separate sexes proved to be the best even for unrelated demographic categories. Both figures combined with Table 2 suggest that even when those two RFs estimate S-LGB percentages for a specific combination of demographic characteristics poorly (e.g., male Hispanic population), the overall median ICC is still above 0.5 across most prediction subgroups. Therefore, the two RFs might be applied selectively to demographic subgroups shown to have a higher prediction capability. Results could support further refinement with techniques such as iterative proportional weighting.

This study’s methodology is flexible enough to adapt for new rounds of YRBS as they are released. Moreover, our estimates could identify opportunities to support the health and well-being needs of youth who identify as LGB across different demographic groups.

## Supporting information

S1 Table Pooled 2021 Youth Risk Behavior Survey respondents from all states along sex, race and ethnicity, and age groups. (DOCX)

S2 Table Median demographic composition of 2021 Youth Risk Behavior Survey respondents among all states for which data are available. (DOCX)

S3 Table Candidate and chosen values when tuning the hyperparameters used in each of the 35 random forest models trained in the Aggregate approach. (DOCX)

S4 Table Comparison between intraclass correlation coefficient (ICC), Total Squared Error and Mean Squared Error of different estimation approaches applied to each prediction subgroup. (DOCX)

S1 Appendix Hyperparameter details. Summarizes the hyperparameter selection process.
(DOCX)

S2 Appendix Metrics resulting from each estimation approach in each prediction subgroup. Data tables and figures with additional results omitted from the main manuscript.
(DOCX)

S1 Fig Charts comparing the observed and predicted S-LGB percentages in each state and sex. (TIFF)

S2 Fig Charts comparing the observed and predicted S-LGB percentages in each state and age. (TIFF)

S3 Fig Charts comparing the observed and predicted S-LGB percentages in each state and race. (TIFF)

S4 Fig Charts comparing the observed and predicted S-LGB percentages in each state, sex, and age. (TIFF)

S5 Fig Charts comparing the observed and predicted S-LGB percentages in each state, sex, and race. (TIFF)

S6 Fig Charts comparing the observed and predicted S-LGB percentages of females in each state, race, and age for the logistic regression. (TIFF)

S7 Fig Charts comparing the observed and predicted S-LGB percentages of females in each state, race, and age for the random forest. (TIFF)

S8 Fig Charts comparing the observed and predicted S-LGB percentages of males in each state, race, and age for the logistic regression. (TIFF)

S9 Fig Charts comparing the observed and predicted S-LGB percentages of males in each state, race, and age for the random forest. (TIFF)

S10 Fig Charts of the estimated S-LGB percentages along states. (TIFF)

S11 Fig Charts of the estimated S-LGB percentages along states and race. (TIFF)

S12 Fig Charts of the estimated S-LGB percentages along states and age. (TIFF)

S13 Fig Charts of the estimated S-LGB percentages along states, sex, and race. (TIFF)

S14 Fig Charts of the estimated S-LGB percentages along states, sex, and age. (TIFF)

S15 Fig Charts of the estimated S-LGB percentages of females along states, race, and age. (TIFF)

S16 Fig Charts of the estimated S-LGB percentages of males along states, race, and age. (TIFF)
