# Supplementary material for: Indirect state-level estimation of sexual minority adolescent populations by sex, age, and race/ethnicity using random forests
Source: PLoS One. 2026 Jun 9;21(6):e0349759. doi: 10.1371/journal.pone.0349759 (PMC13249400; doi:10.1371/journal.pone.0349759)
Supplement: S1 Table — (DOCX) [file pone.0349759.s001.docx]

| Sex | Race and Ethnicity | Age | Respondent N | Respondent % |
| --- | --- | --- | --- | --- |
| Female | Non-Hispanic White | 14yo | 8848 | 6.0% |
|  |  | 15yo | 11,297 | 7.6% |
|  |  | 16yo | 10,134 | 6.9% |
|  |  | 17yo | 8578 | 5.8% |
|  |  | 18+yo | 2004 | 1.4% |
|  | Non-Hispanic Black | 14yo | 2082 | 1.4% |
|  |  | 15yo | 2570 | 1.7% |
|  |  | 16yo | 2215 | 1.5% |
|  |  | 17yo | 1895 | 1.3% |
|  |  | 18+yo | 476 | 0.3% |
|  | Hispanic | 14yo | 3078 | 2.1% |
|  |  | 15yo | 3641 | 2.5% |
|  |  | 16yo | 3109 | 2.1% |
|  |  | 17yo | 2609 | 1.8% |
|  |  | 18+yo | 769 | 0.5% |
|  | Non-Hispanic American Indian/Alaska Native, Asian, Native Hawaiian, Other Pacific Islander, or Non-Hispanic Multiracial | 14yo | 2298 | 1.6% |
|  |  | 15yo | 2596 | 1.8% |
|  |  | 16yo | 2355 | 1.6% |
|  |  | 17yo | 1885 | 1.3% |
|  |  | 18+yo | 399 | 0.3% |
| Male | Non-Hispanic White | 14yo | 8662 | 5.9% |
|  |  | 15yo | 11,885 | 8.0% |
|  |  | 16yo | 10,295 | 7.0% |
|  |  | 17yo | 8928 | 6.0% |
|  |  | 18+yo | 2756 | 1.9% |
|  | Non-Hispanic Black | 14yo | 2025 | 1.4% |
|  |  | 15yo | 2530 | 1.7% |
|  |  | 16yo | 2227 | 1.5% |
|  |  | 17yo | 1856 | 1.3% |
|  |  | 18+yo | 664 | 0.4% |
|  | Hispanic | 14yo | 2845 | 1.9% |
|  |  | 15yo | 3555 | 2.4% |
|  |  | 16yo | 3340 | 2.3% |
|  |  | 17yo | 2720 | 1.8% |
|  |  | 18+yo | 980 | 0.7% |
|  | Non-Hispanic American Indian/Alaska Native, Asian, Native Hawaiian, Other Pacific Islander, or Non-Hispanic Multiracial | 14yo | 2167 | 1.5% |
|  |  | 15yo | 2756 | 1.9% |
|  |  | 16yo | 2340 | 1.6% |
|  |  | 17yo | 1959 | 1.3% |

Abbreviations: yo: year-olds
