# Supplementary material for: Indirect state-level estimation of sexual minority adolescent populations by sex, age, and race/ethnicity using random forests
Source: PLoS One. 2026 Jun 9;21(6):e0349759. doi: 10.1371/journal.pone.0349759 (PMC13249400; doi:10.1371/journal.pone.0349759)
Supplement: S2 Table — (DOCX) [file pone.0349759.s002.docx]

| Sex | Race and Ethnicity | Age | Respondent N | Respondent % |
| --- | --- | --- | --- | --- |
| Female | Non-Hispanic White | 14yo | 81 | 5.1% |
|  |  | 15yo | 122.5 | 7.9% |
|  |  | 16yo | 102.5 | 6.8% |
|  |  | 17yo | 81.5 | 5.4% |
|  |  | 18+yo | 20.5 | 1.5% |
|  | Non-Hispanic Black | 14yo | 19 | 1.0% |
|  |  | 15yo | 25 | 1.2% |
|  |  | 16yo | 21 | 1.0% |
|  |  | 17yo | 11 | 0.7% |
|  |  | 18+yo | 6 | 0.4% |
|  | Hispanic | 14yo | 28 | 1.7% |
|  |  | 15yo | 42.5 | 2.7% |
|  |  | 16yo | 37 | 2.0% |
|  |  | 17yo | 35 | 1.5% |
|  |  | 18+yo | 9 | 0.5% |
|  | Non-Hispanic American Indian/Alaska Native, Asian, Native Hawaiian, Other Pacific Islander, or Non-Hispanic Multiracial | 14yo | 20 | 1.2% |
|  |  | 15yo | 25.5 | 1.4% |
|  |  | 16yo | 18.5 | 1.2% |
|  |  | 17yo | 17 | 1.0% |
|  |  | 18+yo | 5 | 0.3% |
| Male | Non-Hispanic White | 14yo | 74 | 4.6% |
|  |  | 15yo | 114 | 7.6% |
|  |  | 16yo | 103.5 | 6.6% |
|  |  | 17yo | 95.5 | 5.4% |
|  |  | 18+yo | 30.5 | 1.8% |
|  | Non-Hispanic Black | 14yo | 19.5 | 1.0% |
|  |  | 15yo | 28 | 1.4% |
|  |  | 16yo | 22 | 1.2% |
|  |  | 17yo | 16 | 0.8% |
|  |  | 18+yo | 9 | 0.4% |
|  | Hispanic | 14yo | 28 | 1.5% |
|  |  | 15yo | 42 | 2.2% |
|  |  | 16yo | 39 | 2.1% |
|  |  | 17yo | 31 | 1.8% |
|  |  | 18+yo | 13 | 0.6% |
|  | Non-Hispanic American Indian/Alaska Native, Asian, Native Hawaiian, Other Pacific Islander, or Non-Hispanic Multiracial | 14yo | 18.5 | 1.1% |
|  |  | 15yo | 23.5 | 1.3% |
|  |  | 16yo | 21 | 1.2% |
|  |  | 17yo | 17.5 | 1.0% |
|  |  | 18+yo | 6.5 | 0.3% |

Abbreviations: yo: year-olds
