# Supplementary material for: Indirect state-level estimation of sexual minority adolescent populations by sex, age, and race/ethnicity using random forests
Source: PLoS One. 2026 Jun 9;21(6):e0349759. doi: 10.1371/journal.pone.0349759 (PMC13249400; doi:10.1371/journal.pone.0349759)
Supplement: S3 Table — (DOCX) [file pone.0349759.s003.docx]

| Hyperparameter | Candidate Values | Chosen Value for Aggregate Approach |
| --- | --- | --- |
| Number of random trees trained in each forest | 50, 100, 150, 200 | 150 |
| Maximum depth for each random tree | 10, 12, 14, 16 | 14 |
| Minimum samples accepted in a node | 2, 5, 10, 15 | 15 |
| Maximum features considered to split a node | #*parameters/4,* #*parameters/3,* #*parameters/2* | #*parameters/3* |
| Bootstrap samples to promote generalization | Yes or No | Yes |

Note: We tested a random subset of 100 possible combinations of the hyperparameters shown in the second column for the highest Intraclass Correlation Coefficient after Leave-One-Group-Out validation. The chosen combination is shown in the rightmost column.
