# Supplementary material for: Indirect state-level estimation of sexual minority adolescent populations by sex, age, and race/ethnicity using random forests
Source: PLoS One. 2026 Jun 9;21(6):e0349759. doi: 10.1371/journal.pone.0349759 (PMC13249400; doi:10.1371/journal.pone.0349759)
Supplement: S4 Table — (DOCX) [file pone.0349759.s004.docx]

| Prediction Subgroup | Estimation approach | ICC | Total Error | Mean Error |
| --- | --- | --- | --- | --- |
| Statewide | Logistic regression (LR) on Aggregate | −0.21149 | 2333.187 | 0.013295 |
|  | Random forest (RF) on Aggregate | 0.709066 | 2491.19 | 0.014195 |
|  | RF on datasets separated by sex (*S^x^*) | 0.751033 | 2424.389 | 0.013815 |
|  | RF on datasets separated by race (*S^e^*) | 0.668963 | 2886.046 | 0.016445 |
|  | RF on datasets separated by age (*S^a^*) | 0.709627 | 2950.518 | 0.016813 |
|  | RF on datasets separated by sex and race (*S^xe^*) | 0.673688 | 2941.769 | 0.016763 |
|  | RF on datasets separated by sex and age (*S^xa^*) | 0.715093 | 3146.643 | 0.01793 |
|  | RF on datasets separated by race and age (*S^ea^*) | 0.571869 | 4057.719 | 0.023122 |
| Each sex | LR on Aggregate | −0.12907 | 2709.563 | 0.01544 |
|  | RF on Aggregate | 0.704444 | 2716.085 | 0.015477 |
|  | RF on *S^x^* | 0.733406 | 2680.441 | 0.015274 |
|  | RF on *S^e^* | 0.669528 | 3217.835 | 0.018336 |
|  | RF on *S^a^* | 0.684634 | 3273.897 | 0.018655 |
|  | RF on *S^xe^* | 0.670057 | 3289.071 | 0.018742 |
|  | RF on *S^ea^* | 0.682678 | 3424.624 | 0.019514 |
|  | RF on *S^ea^* | 0.555144 | 4479.812 | 0.025527 |
| Racial groups | LR on Aggregate | −0.1632 | 3232.166 | 0.018299 |
|  | RF on Aggregate | 0.573112 | 2822.048 | 0.015977 |
|  | RF on *S^x^* | 0.610655 | 2769.853 | 0.015682 |
|  | RF on *S^e^* | 0.59913 | 3156.998 | 0.017874 |
|  | RF on *S^a^* | 0.567586 | 3189.345 | 0.018057 |
|  | RF on *S^xe^* | 0.608556 | 3297.546 | 0.018669 |
|  | RF on *S^xa^* | 0.588126 | 3273.521 | 0.018533 |
|  | RF on *S^ea^* | 0.556237 | 4211.183 | 0.023842 |
| Age groups | LR on Aggregate | −0.04972 | 3767.005 | 0.021076 |
|  | RF on Aggregate | 0.658716 | 3389.233 | 0.018962 |
|  | RF on *S^x^* | 0.677056 | 3350.772 | 0.018747 |
|  | RF on *S^e^* | 0.625552 | 3707.427 | 0.020743 |
|  | RF on *S^a^* | 0.598096 | 3988.199 | 0.022313 |
|  | RF on *S^xe^* | 0.626501 | 3812.758 | 0.021332 |
|  | RF on *S^xa^* | 0.599388 | 4090.839 | 0.022888 |
|  | RF on *S^ea^* | 0.493952 | 4765.419 | 0.026662 |
| Sex/race | LR on Aggregate | −0.06603 | 4053.041 | 0.023005 |
|  | RF on Aggregate | 0.591288 | 3547.169 | 0.020133 |
|  | RF on *S^x^* | 0.602506 | 3505.364 | 0.019896 |
|  | RF on *S^e^* | 0.598252 | 3962.12 | 0.022489 |
|  | RF on *S^a^* | 0.564695 | 4063.277 | 0.023063 |
|  | RF on *S^xe^* | 0.606756 | 4043.777 | 0.022952 |
|  | RF on *S^xa^* | 0.577416 | 4107.613 | 0.023315 |
|  | RF on *S^ea^* | 0.508031 | 4943.893 | 0.028061 |
| Sex/age | LR on Aggregate | −0.05472 | 4826.004 | 0.026928 |
|  | RF on Aggregate | 0.599391 | 4413.485 | 0.024627 |
|  | RF on *S^x^* | 0.61496 | 4386.419 | 0.024476 |
|  | RF on *S^e^* | 0.573125 | 4803.794 | 0.026805 |
|  | RF on *S^a^* | 0.549995 | 5079.93 | 0.028345 |
|  | RF on *S^xe^* | 0.573428 | 4823.294 | 0.026913 |
|  | RF on *S^xa^* | 0.558384 | 5072.401 | 0.028303 |
|  | RF on *S^ea^* | 0.461211 | 5945.471 | 0.033175 |
| Race/age | LR on Aggregate | 0.118887 | 5512.186 | 0.031829 |
|  | RF on Aggregate | 0.559355 | 4841.799 | 0.027958 |
|  | RF on *S^x^* | 0.575464 | 4827.124 | 0.027874 |
|  | RF on *S^e^* | 0.556407 | 5145.418 | 0.029712 |
|  | RF on *S^a^* | 0.513003 | 5383.387 | 0.031086 |
|  | RF on *S^xe^* | 0.563838 | 5213.759 | 0.030106 |
|  | RF on *S^xa^* | 0.518882 | 5353.462 | 0.030913 |
|  | RF on *S^ea^* | 0.479852 | 6051.858 | 0.034946 |
| Sex/race/age | LR on Aggregate | −0.0257 | 7492.635 | 0.042917 |
|  | RF on Aggregate | 0.462502 | 6579.666 | 0.037688 |
|  | RF on *S^x^* | 0.465962 | 6572.315 | 0.037646 |
|  | RF on *S^e^* | 0.451908 | 6828.859 | 0.039115 |
|  | RF on *S^a^* | 0.406298 | 7071.467 | 0.040505 |
|  | RF on *S^xe^* | 0.459722 | 6859.664 | 0.039291 |
|  | RF on *S^xa^* | 0.414094 | 7035.3 | 0.040298 |
|  | RF on *S^ea^* | 0.367858 | 7746.582 | 0.044372 |

Note: *S^x^* is the estimation approach in which 2 random forests are trained, one for each sex; *S^a^* and *S^e^* are analogous to *S^x^*, but for 5 random forests trained in different age groups, and 4 random forests trained on different race and ethnicities, respectively. *S^xe^*, *S^xa^* and *S^ea^* refer to the estimation approaches of training multiple random forests, each trained on a group of respondents with intersecting demographic characteristics.
