# Supplementary material for: Indirect state-level estimation of sexual minority adolescent populations by sex, age, and race/ethnicity using random forests
Source: PLoS One. 2026 Jun 9;21(6):e0349759. doi: 10.1371/journal.pone.0349759 (PMC13249400; doi:10.1371/journal.pone.0349759)
Supplement: S1 Appendix — Summarizes the hyperparameter selection process. (DOCX) [file pone.0349759.s005.docx]

# S1 Appendix. Hyperparameter details

The hyperparameter tuning process considered a subset of five of the available hyperparameters accepted by the *RandomForestClassifier* implementation in Python. For each of those hyperparameters, a limited number of candidate values were arbitrarily chosen based on well-performing hyperparameters values from previous research [8]. S3 Table shows all considered hyperparameters, their candidate values, and ultimately chosen values for the Aggregate estimation approach in 2021. In every repetition, a random value is chosen for each of the hyperparameters. Those values are then used to configure the random forest (RF) models that estimate the S-LGB probability of all respondents within each state, following the same leave-one-group-out (LOGO) validation described in our methods. The combination of random hyperparameter values that results in the best intraclass correlation coefficient (ICC) within the 100 repetitions is chosen as the (locally) optimal combination of hyperparameters. We assume that combination to be acceptable for RFs trained not only on the overall population, but also for specialized models with narrower estimation approaches.

**S3 Table. Candidate and chosen values when tuning the hyperparameters used in each of the 35 random forest models trained in the Aggregate approach.**

| Hyperparameter | Candidate Values | Chosen Value for Aggregate Approach |
| --- | --- | --- |
| Number of random trees trained in each forest | 50, 100, 150, 200 | 150 |
| Maximum depth for each random tree | 10, 12, 14, 16 | 14 |
| Minimum samples accepted in a node | 2, 5, 10, 15 | 15 |
| Maximum features considered to split a node | #*parameters/4,* #*parameters/3,* #*parameters/2* | #*parameters/3* |
| Bootstrap samples to promote generalization | Yes or No | Yes |

Note: We tested a random subset of 100 possible combinations of the hyperparameters shown in the second column for the highest Intraclass Correlation Coefficient after Leave-One-Group-Out validation. The chosen combination is shown in the rightmost column.

## Variable Coding

One-hot encoding was used to recode the inputs of all tested RFs. Most YRBS questions have categorical answers with no ordinality and are thus recoded into binary variables representing each answer. The few continuous features retained include weight and height of the respondent, as well as the corresponding body mass index (BMI) and comparative BMI percentages.

# Appendix C

## Metrics resulting from each estimation approach in each prediction subgroup

**S4 Table. Comparison between intraclass correlation coefficient (ICC), Total Squared Error and Mean Squared Error of different estimation approaches applied to each prediction subgroup.**

| Prediction Subgroup | Estimation approach | ICC | Total Error | Mean Error |
| --- | --- | --- | --- | --- |
| Statewide | Logistic regression (LR) on Aggregate | −0.21149 | 2333.187 | 0.013295 |
|  | Random forest (RF) on Aggregate | 0.709066 | 2491.19 | 0.014195 |
|  | RF on datasets separated by sex (*S^x^*) | 0.751033 | 2424.389 | 0.013815 |
|  | RF on datasets separated by race (*S^e^*) | 0.668963 | 2886.046 | 0.016445 |
|  | RF on datasets separated by age (*S^a^*) | 0.709627 | 2950.518 | 0.016813 |
|  | RF on datasets separated by sex and race (*S^xe^*) | 0.673688 | 2941.769 | 0.016763 |
|  | RF on datasets separated by sex and age (*S^xa^*) | 0.715093 | 3146.643 | 0.01793 |
|  | RF on datasets separated by race and age (*S^ea^*) | 0.571869 | 4057.719 | 0.023122 |
| Each sex | LR on Aggregate | −0.12907 | 2709.563 | 0.01544 |
|  | RF on Aggregate | 0.704444 | 2716.085 | 0.015477 |
|  | RF on *S^x^* | 0.733406 | 2680.441 | 0.015274 |
|  | RF on *S^e^* | 0.669528 | 3217.835 | 0.018336 |
|  | RF on *S^a^* | 0.684634 | 3273.897 | 0.018655 |
|  | RF on *S^xe^* | 0.670057 | 3289.071 | 0.018742 |
|  | RF on *S^ea^* | 0.682678 | 3424.624 | 0.019514 |
|  | RF on *S^ea^* | 0.555144 | 4479.812 | 0.025527 |
| Racial groups | LR on Aggregate | −0.1632 | 3232.166 | 0.018299 |
|  | RF on Aggregate | 0.573112 | 2822.048 | 0.015977 |
|  | RF on *S^x^* | 0.610655 | 2769.853 | 0.015682 |
|  | RF on *S^e^* | 0.59913 | 3156.998 | 0.017874 |
|  | RF on *S^a^* | 0.567586 | 3189.345 | 0.018057 |
|  | RF on *S^xe^* | 0.608556 | 3297.546 | 0.018669 |
|  | RF on *S^xa^* | 0.588126 | 3273.521 | 0.018533 |
|  | RF on *S^ea^* | 0.556237 | 4211.183 | 0.023842 |
| Age groups | LR on Aggregate | −0.04972 | 3767.005 | 0.021076 |
|  | RF on Aggregate | 0.658716 | 3389.233 | 0.018962 |
|  | RF on *S^x^* | 0.677056 | 3350.772 | 0.018747 |
|  | RF on *S^e^* | 0.625552 | 3707.427 | 0.020743 |
|  | RF on *S^a^* | 0.598096 | 3988.199 | 0.022313 |
|  | RF on *S^xe^* | 0.626501 | 3812.758 | 0.021332 |
|  | RF on *S^xa^* | 0.599388 | 4090.839 | 0.022888 |
|  | RF on *S^ea^* | 0.493952 | 4765.419 | 0.026662 |
| Sex/race | LR on Aggregate | −0.06603 | 4053.041 | 0.023005 |
|  | RF on Aggregate | 0.591288 | 3547.169 | 0.020133 |
|  | RF on *S^x^* | 0.602506 | 3505.364 | 0.019896 |
|  | RF on *S^e^* | 0.598252 | 3962.12 | 0.022489 |
|  | RF on *S^a^* | 0.564695 | 4063.277 | 0.023063 |
|  | RF on *S^xe^* | 0.606756 | 4043.777 | 0.022952 |
|  | RF on *S^xa^* | 0.577416 | 4107.613 | 0.023315 |
|  | RF on *S^ea^* | 0.508031 | 4943.893 | 0.028061 |
| Sex/age | LR on Aggregate | −0.05472 | 4826.004 | 0.026928 |
|  | RF on Aggregate | 0.599391 | 4413.485 | 0.024627 |
|  | RF on *S^x^* | 0.61496 | 4386.419 | 0.024476 |
|  | RF on *S^e^* | 0.573125 | 4803.794 | 0.026805 |
|  | RF on *S^a^* | 0.549995 | 5079.93 | 0.028345 |
|  | RF on *S^xe^* | 0.573428 | 4823.294 | 0.026913 |
|  | RF on *S^xa^* | 0.558384 | 5072.401 | 0.028303 |
|  | RF on *S^ea^* | 0.461211 | 5945.471 | 0.033175 |
| Race/age | LR on Aggregate | 0.118887 | 5512.186 | 0.031829 |
|  | RF on Aggregate | 0.559355 | 4841.799 | 0.027958 |
|  | RF on *S^x^* | 0.575464 | 4827.124 | 0.027874 |
|  | RF on *S^e^* | 0.556407 | 5145.418 | 0.029712 |
|  | RF on *S^a^* | 0.513003 | 5383.387 | 0.031086 |
|  | RF on *S^xe^* | 0.563838 | 5213.759 | 0.030106 |
|  | RF on *S^xa^* | 0.518882 | 5353.462 | 0.030913 |
|  | RF on *S^ea^* | 0.479852 | 6051.858 | 0.034946 |
| Sex/race/age | LR on Aggregate | −0.0257 | 7492.635 | 0.042917 |
|  | RF on Aggregate | 0.462502 | 6579.666 | 0.037688 |
|  | RF on *S^x^* | 0.465962 | 6572.315 | 0.037646 |
|  | RF on *S^e^* | 0.451908 | 6828.859 | 0.039115 |
|  | RF on *S^a^* | 0.406298 | 7071.467 | 0.040505 |
|  | RF on *S^xe^* | 0.459722 | 6859.664 | 0.039291 |
|  | RF on *S^xa^* | 0.414094 | 7035.3 | 0.040298 |
|  | RF on *S^ea^* | 0.367858 | 7746.582 | 0.044372 |

Note: *S^x^* is the estimation approach in which 2 random forests are trained, one for each sex; *S^a^* and *S^e^* are analogous to *S^x^*, but for 5 random forests trained in different age groups, and 4 random forests trained on different race and ethnicities, respectively. *S^xe^*, *S^xa^* and *S^ea^* refer to the estimation approaches of training multiple random forests, each trained on a group of respondents with intersecting demographic characteristics.

## S1 Fig. Charts comparing the observed and predicted S-LGB percentages in each state and sex.

## S2 Fig. Charts comparing the observed and predicted S-LGB percentages in each state and age.

## S3 Fig. Charts comparing the observed and predicted S-LGB percentages in each state and race.

## S4 Fig. Charts comparing the observed and predicted S-LGB percentages in each state, sex, and age.

## S5 Fig. Charts comparing the observed and predicted S-LGB percentages in each state, sex, and race.

## S6 Fig. Charts comparing the observed and predicted S-LGB percentages of females in each state, race, and age for the logistic regression.

## S7 Fig. Charts comparing the observed and predicted S-LGB percentages of females in each state, race, and age for the random forest.

## S8 Fig. Charts comparing the observed and predicted S-LGB percentages of males in each state, race, and age for the logistic regression.

## S9 Fig. Charts comparing the observed and predicted S-LGB percentages of males in each state, race, and age for the random forest.

Abbreviations: YRBS is the state-conducted Youth Risk Behavior Survey.

ICC is the intraclass correlation coefficient across all states calculated between estimates and observed values.

S-LGB are the respondents who would self-identify as lesbian, gay, or bisexual if the sexual identity question were asked in their state YRBS.

“Other” is an abbreviated category that encompasses Non-Hispanic American Indian/Alaska Native, Asian, Native Hawaiian, Other Pacific Islander, or Non-Hispanic Multiracial.

yo corresponds to year-olds.

## S10 Fig. Charts of the estimated S-LGB percentages along states.

## S11 Fig. Charts of the estimated S-LGB percentages along states and race.

## S12 Fig. Charts of the estimated S-LGB percentages along states and age.

## S13 Fig. Charts of the estimated S-LGB percentages along states, sex, and race.

## S14 Fig. Charts of the estimated S-LGB percentages along states, sex, and age.

## S15 Fig. Charts of the estimated S-LGB percentages of females along states, race, and age.

## S16 Fig. Charts of the estimated S-LGB percentages of males along states, race, and age.

Note: For S15 and S16 Figs, some of the lower error bounds fall below 0% due to the assumption of a normal distribution of the errors. In the figures, all values below 0% were truncated. All values are percentages between 0 and 1. In S13 Fig, female respondents always have a higher percentage than male respondents in each given state.

Abbreviations: YRBS is the state-conducted Youth Risk Behavior Survey.

LGB means lesbian, gay, or bisexual

S-LGB are the respondents who would self-identify as lesbian, gay, or bisexual if the sexual identity question were asked in their state YRBS.

LGB-N are the states that either did not include or have data available for the sexual identity question in their 2021 YRBS questionnaire

“Other” is an abbreviated category that encompasses Non-Hispanic American Indian/Alaska Native, Asian, Native Hawaiian, Other Pacific Islander, or Non-Hispanic Multiracial.

yo corresponds to year-olds.
