# Supplementary figures and images for: Indirect state-level estimation of sexual minority adolescent populations by sex, age, and race/ethnicity using random forests
Source: PLoS One. 2026 Jun 9;21(6):e0349759. doi: 10.1371/journal.pone.0349759 (PMC13249400; doi:10.1371/journal.pone.0349759)

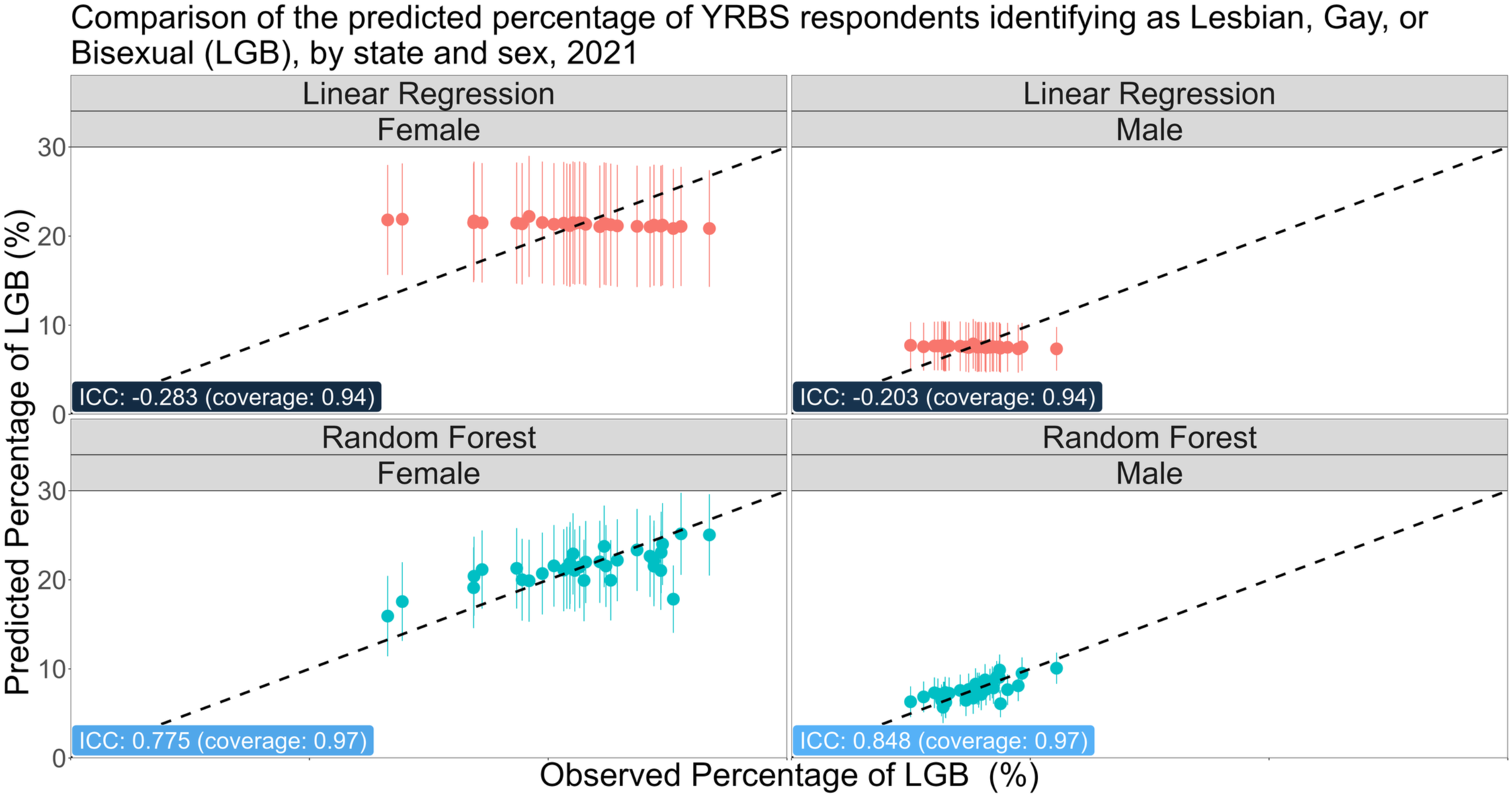

Supplement: S1 Fig — (TIFF) [file pone.0349759.s007.tiff]

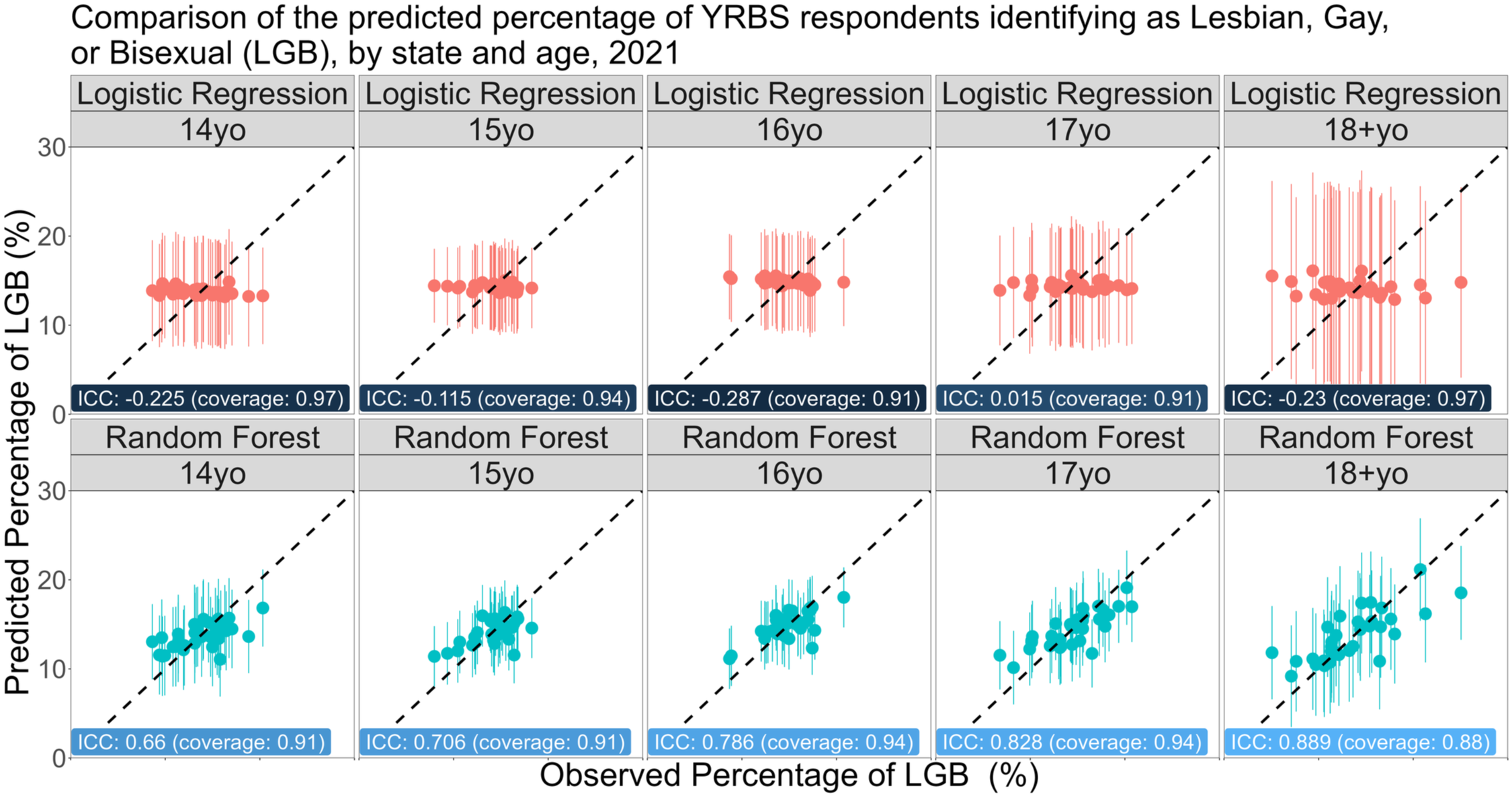

Supplement: S2 Fig — (TIFF) [file pone.0349759.s008.tiff]

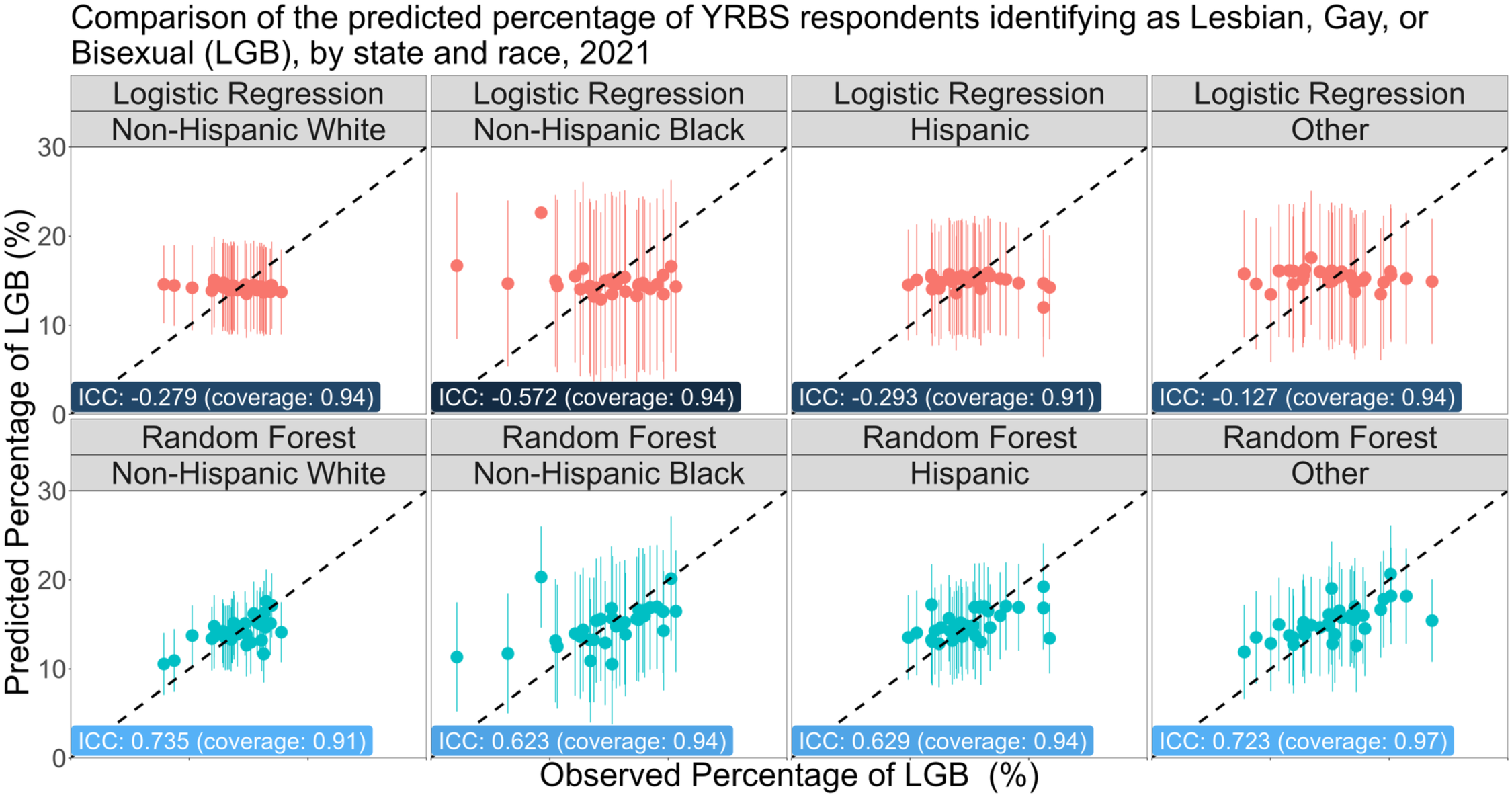

Supplement: S3 Fig — (TIFF) [file pone.0349759.s009.tiff]

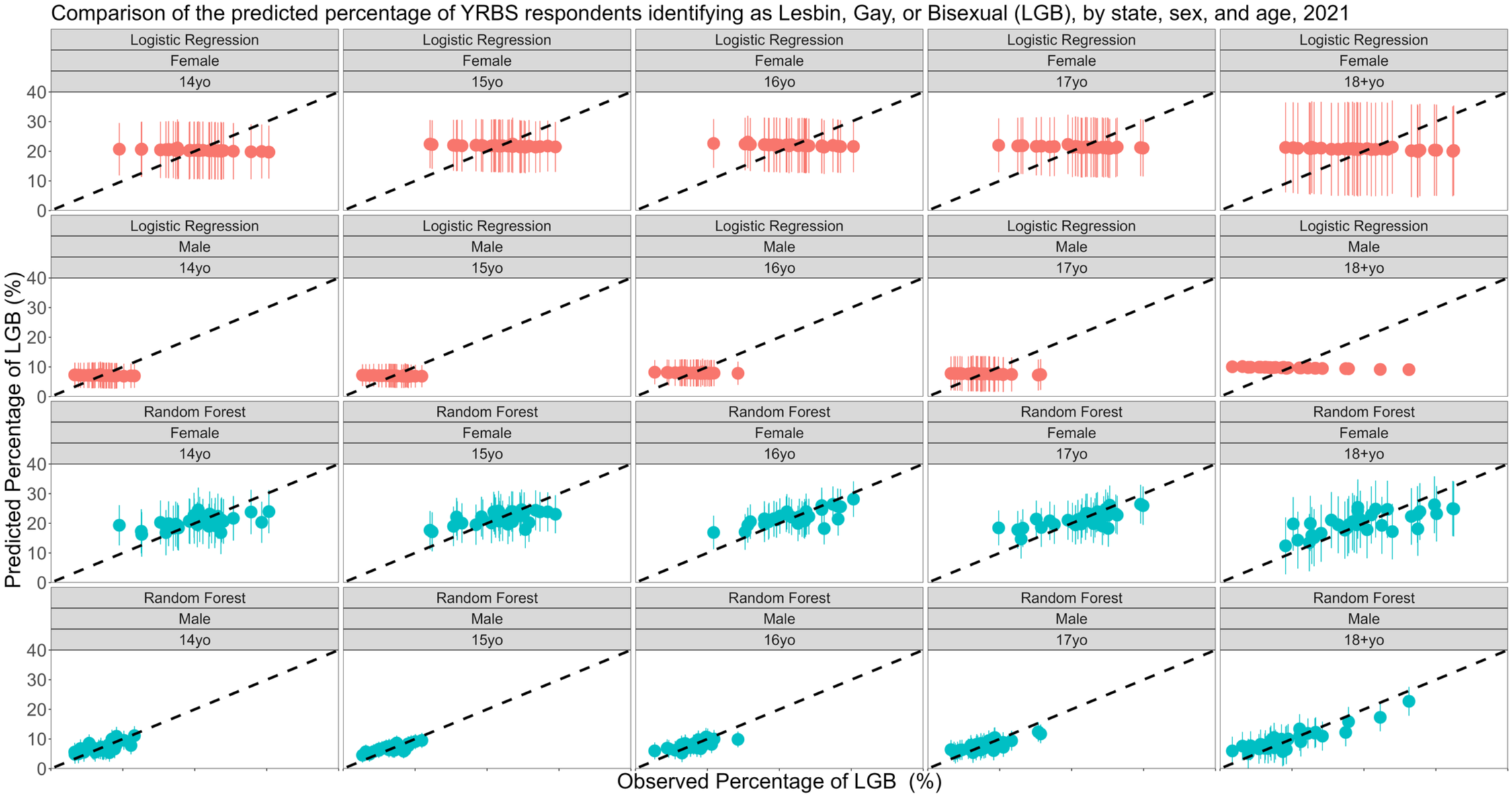

Supplement: S4 Fig — (TIFF) [file pone.0349759.s010.tiff]

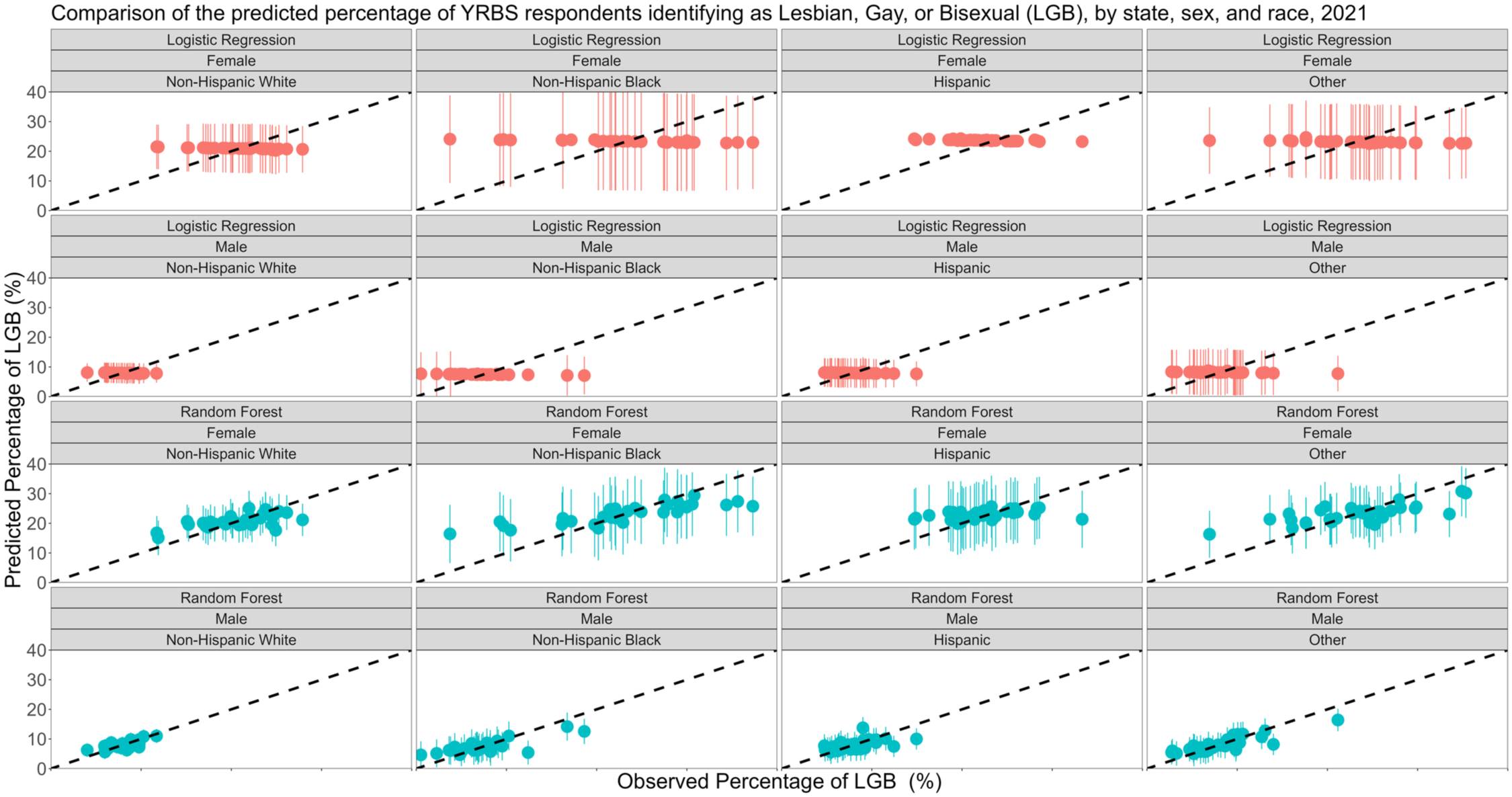

Supplement: S5 Fig — (TIFF) [file pone.0349759.s011.tiff]

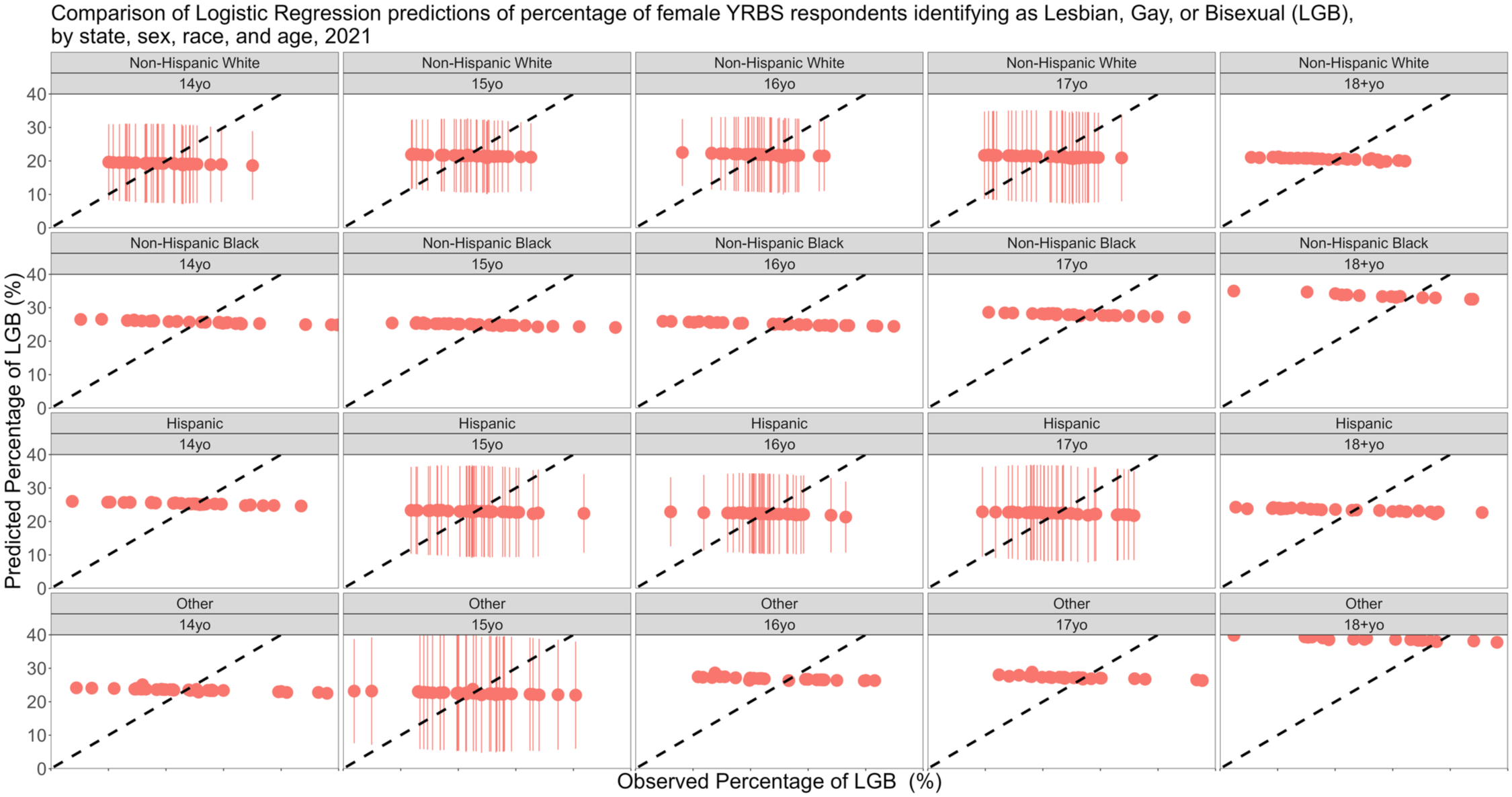

Supplement: S6 Fig — (TIFF) [file pone.0349759.s012.tiff]

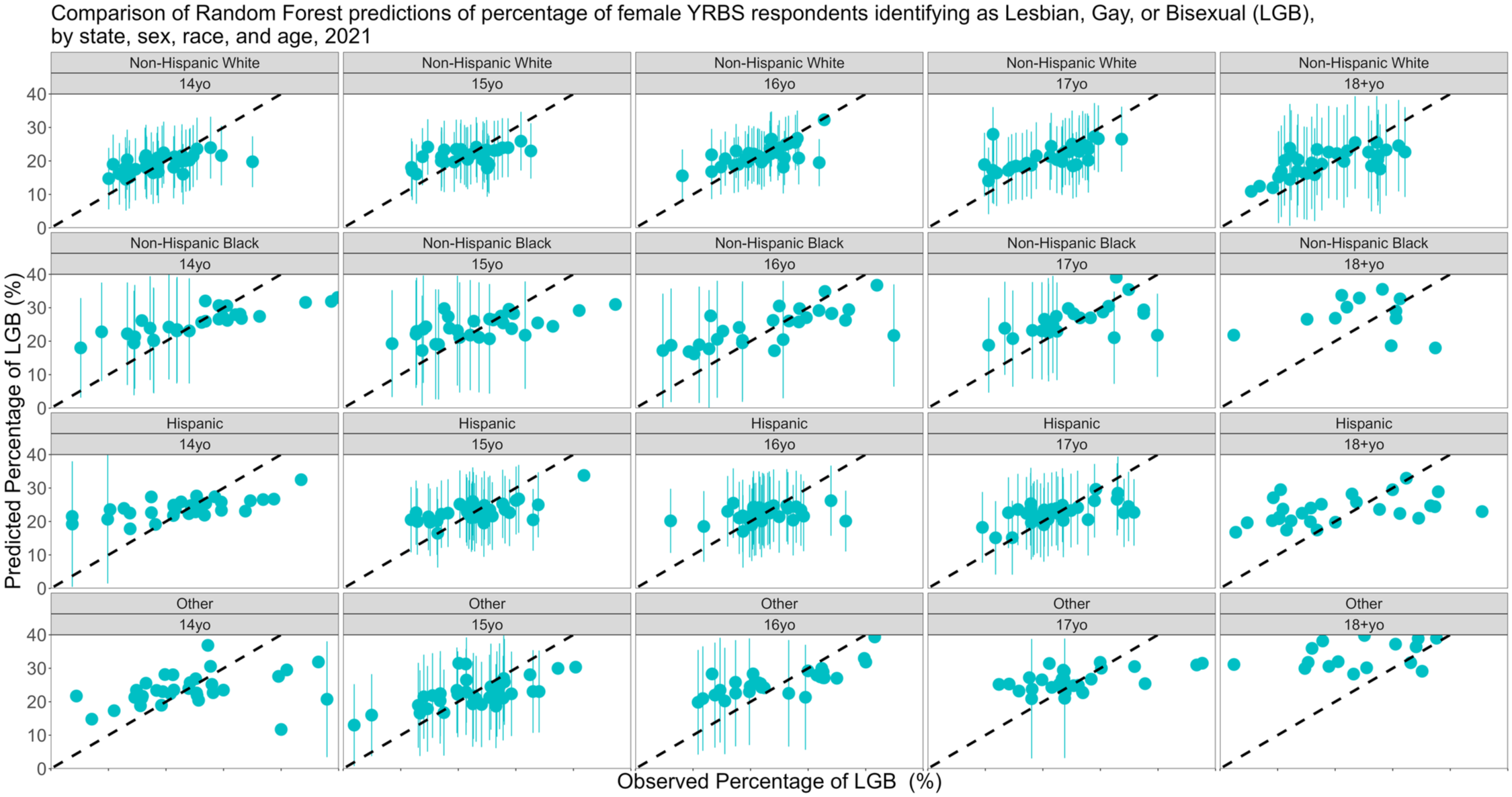

Supplement: S7 Fig — (TIFF) [file pone.0349759.s013.tiff]

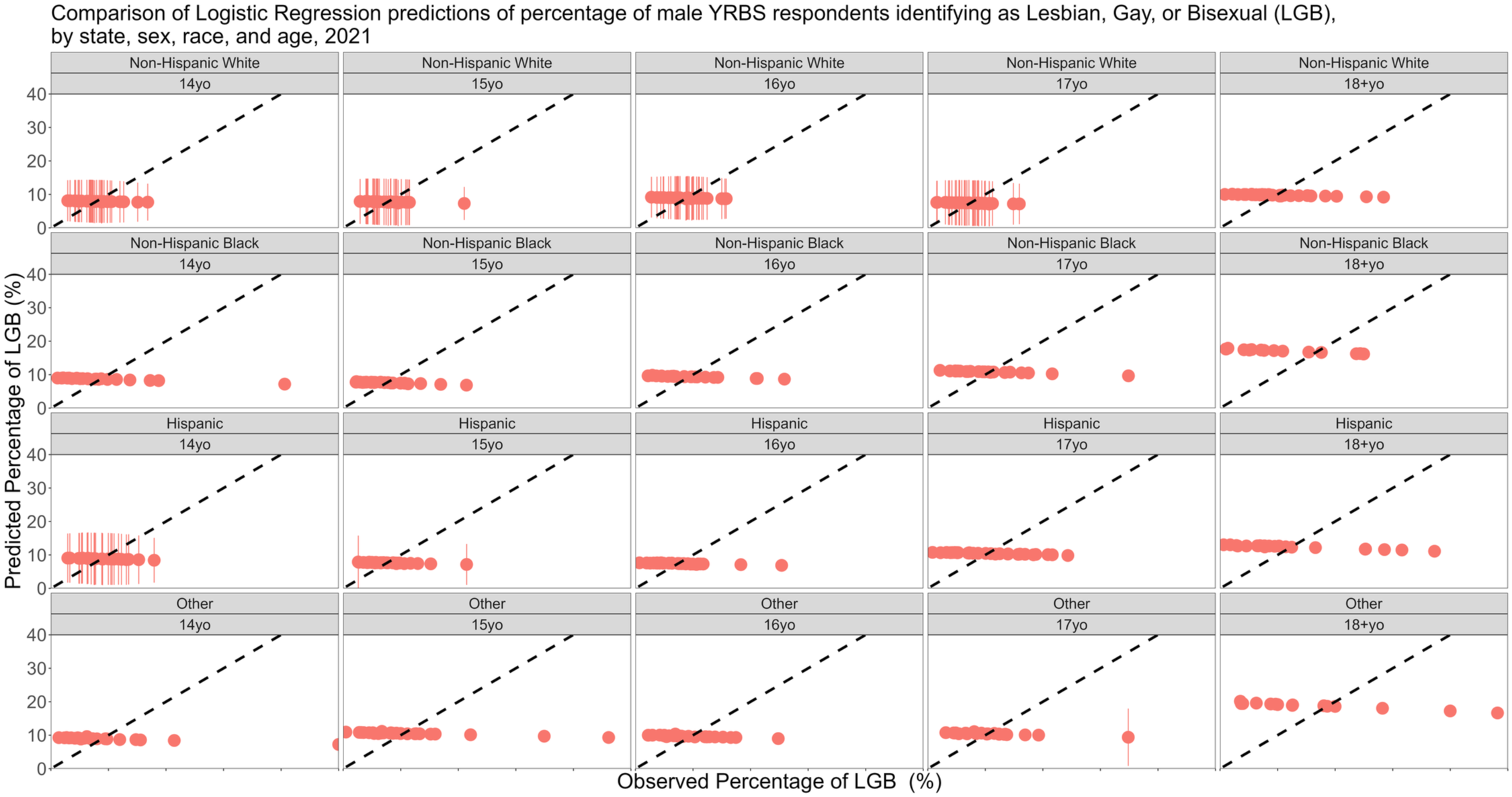

Supplement: S8 Fig — (TIFF) [file pone.0349759.s014.tiff]

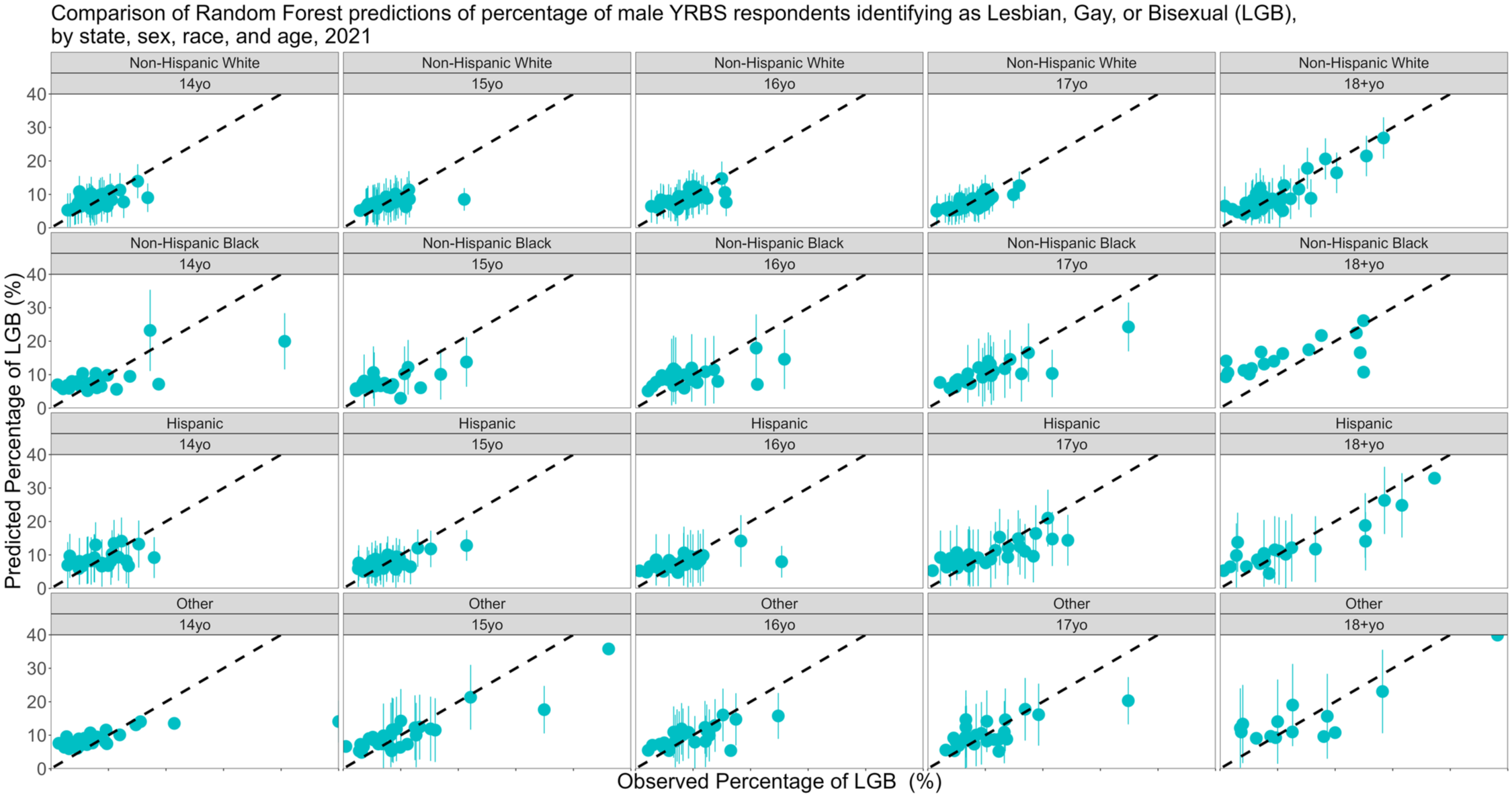

Supplement: S9 Fig — (TIFF) [file pone.0349759.s015.tiff]

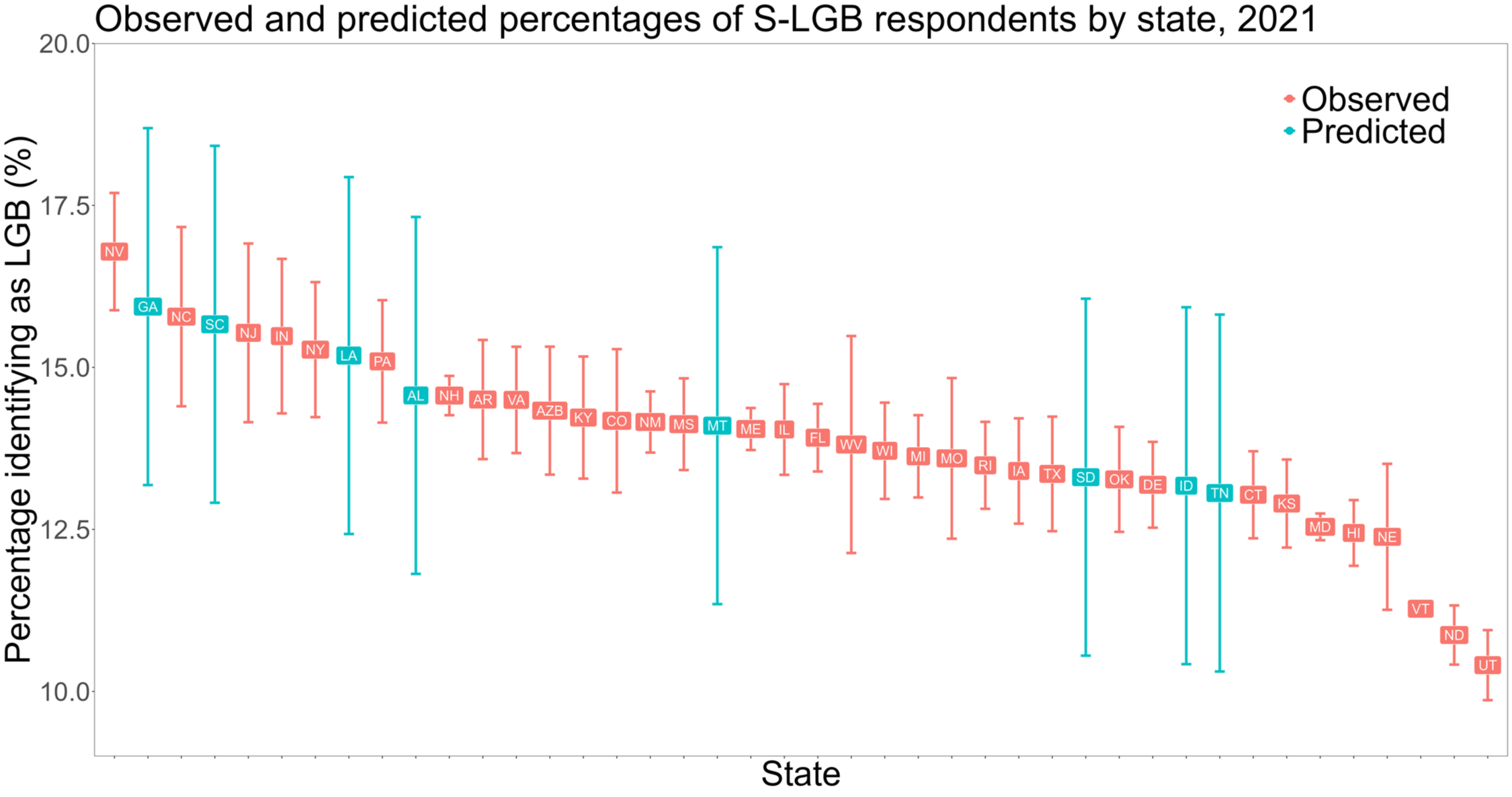

Supplement: S10 Fig — (TIFF) [file pone.0349759.s016.tiff]

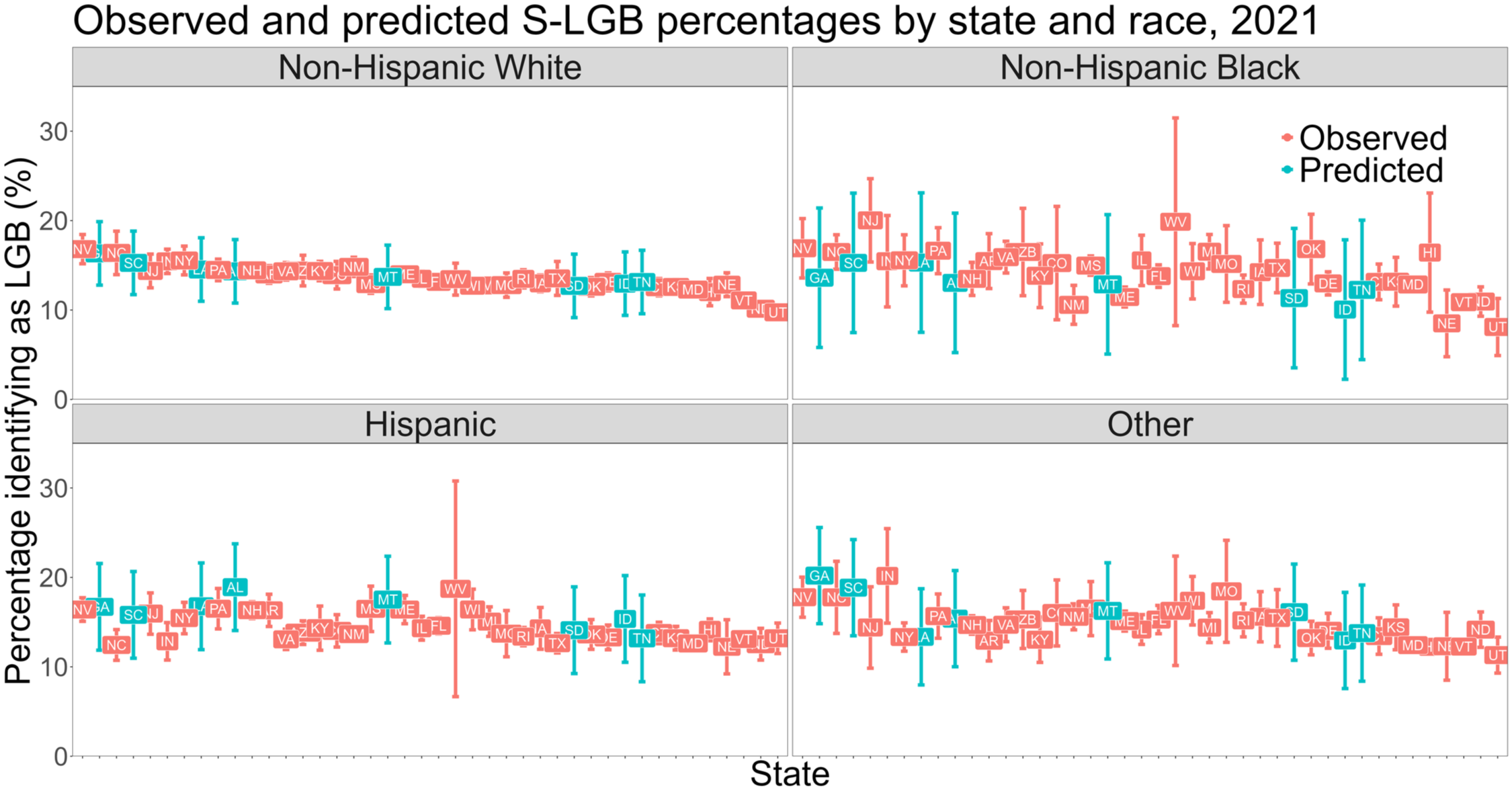

Supplement: S11 Fig — (TIFF) [file pone.0349759.s017.tiff]

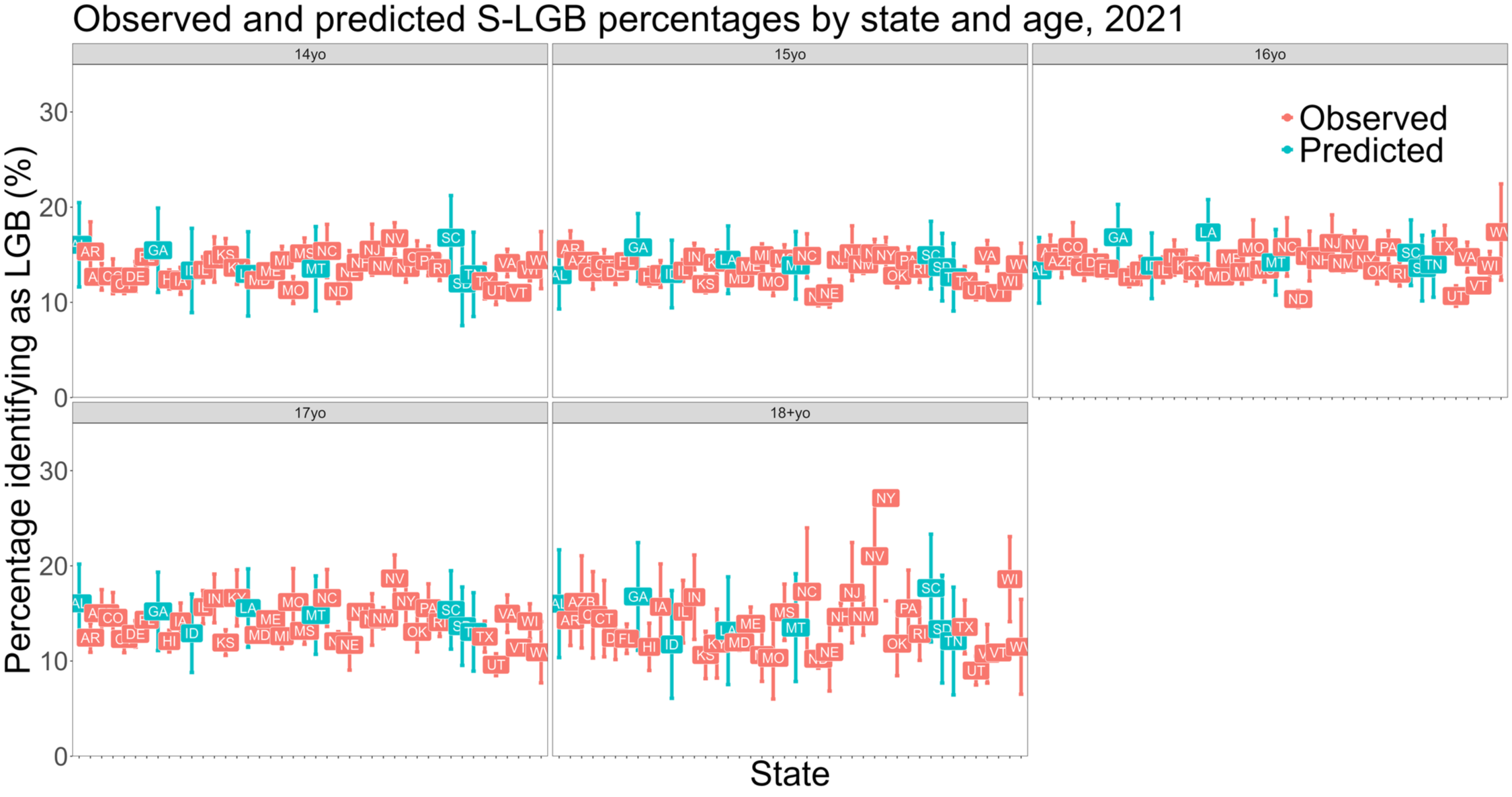

Supplement: S12 Fig — (TIFF) [file pone.0349759.s018.tiff]

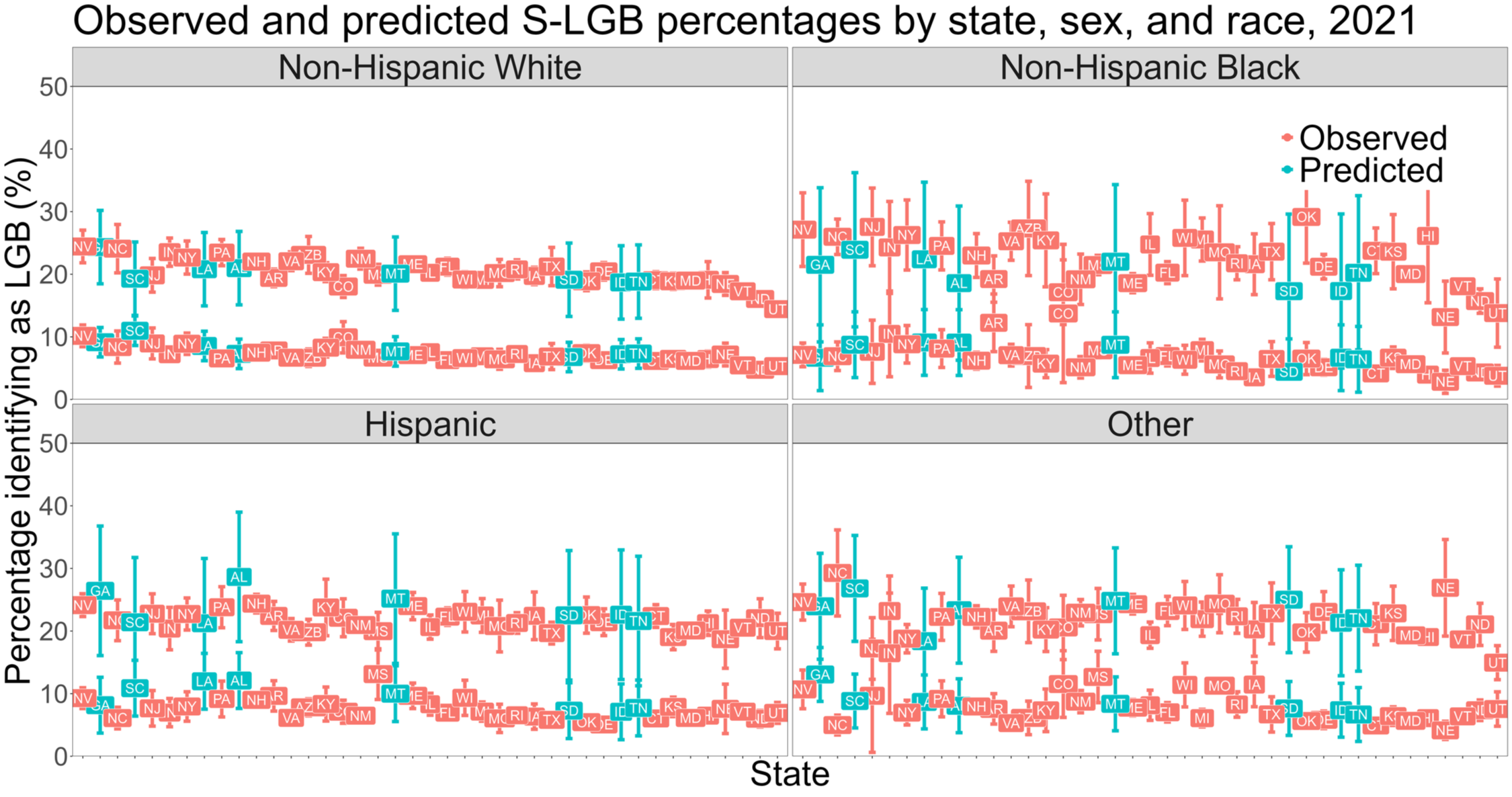

Supplement: S13 Fig — (TIFF) [file pone.0349759.s019.tiff]

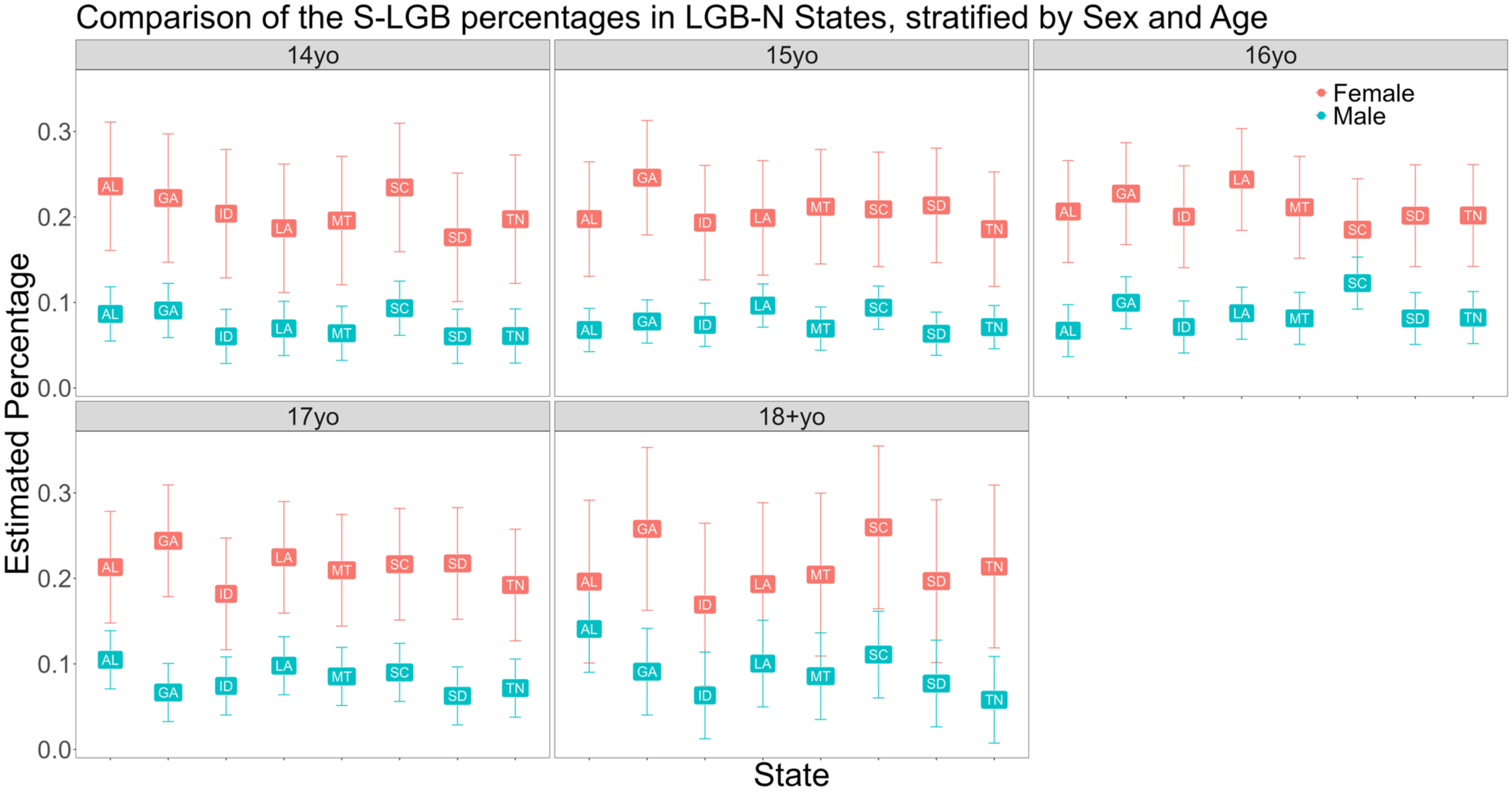

Supplement: S14 Fig — (TIFF) [file pone.0349759.s020.tiff]

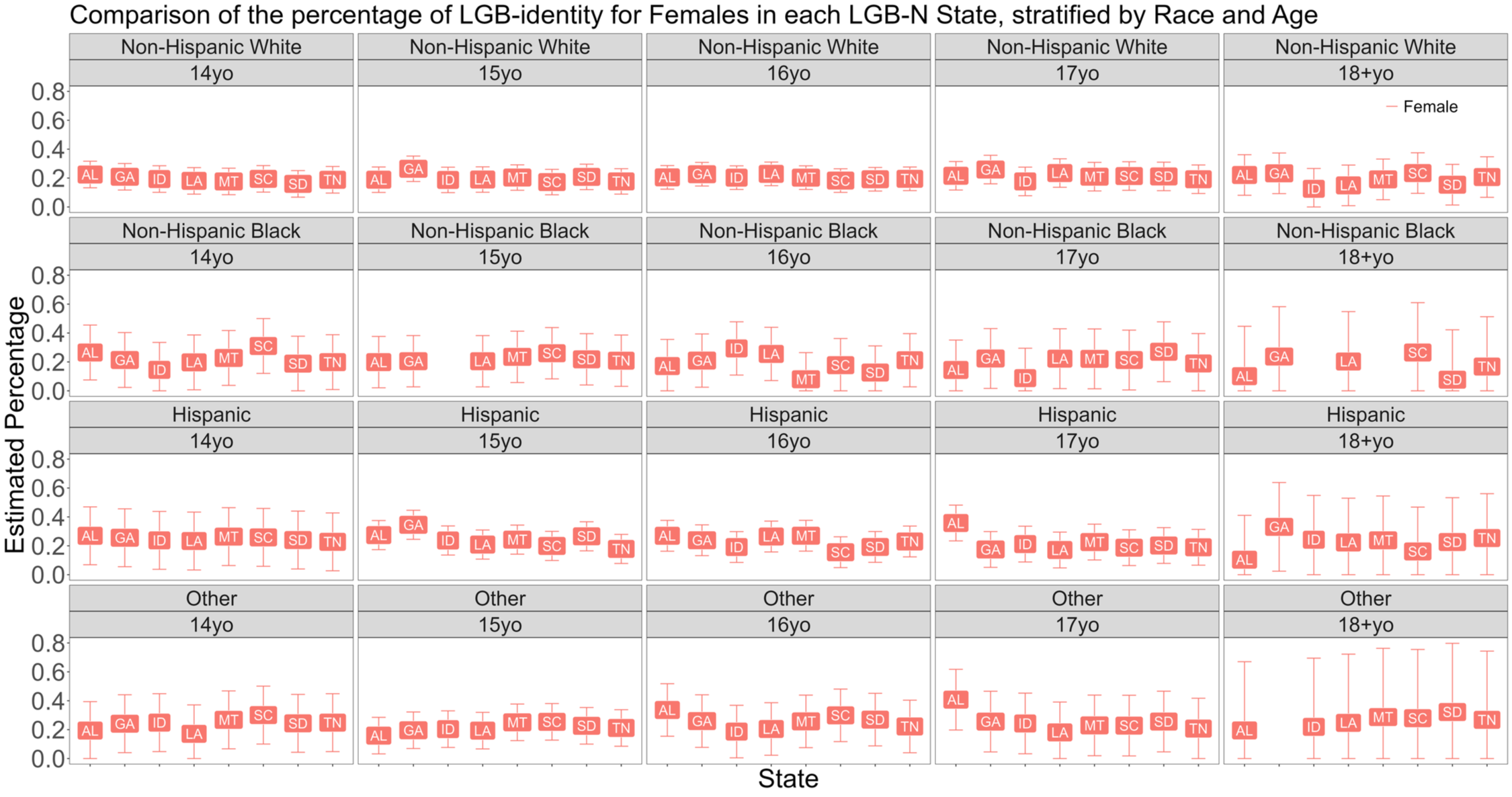

Supplement: S15 Fig — (TIFF) [file pone.0349759.s021.tiff]

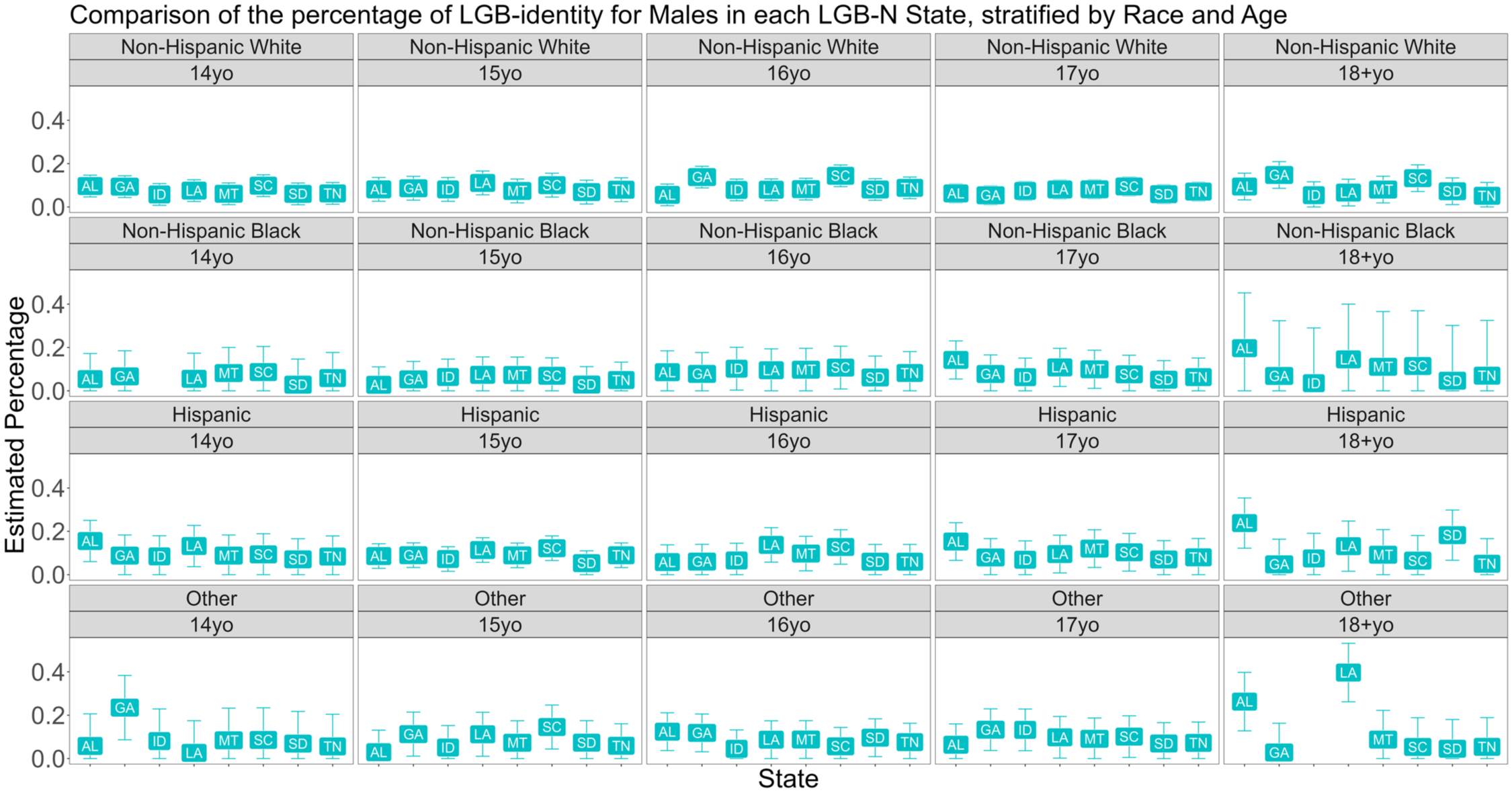

Supplement: S16 Fig — (TIFF) [file pone.0349759.s022.tiff]
