# Supplementary material for: Indirect state-level estimation of sexual minority adolescent populations by sex, age, and race/ethnicity using random forests
Source: PLoS One. 2026 Jun 9;21(6):e0349759. doi: 10.1371/journal.pone.0349759 (PMC13249400; doi:10.1371/journal.pone.0349759)
Supplement: Attachment — Submitted filename: Response to Reviewers.docx [file pone.0349759.s024.docx]

Dear editor and reviewers,

Thank you for reading our manuscript and offering constructive feedback that helps improve our work and better communicate our findings. We have prepared this letter as a response to all concerns and suggestions raised during the reviewing process. We believe the revised version of our manuscript addresses all concerns previously raised and would appreciate any further comments or feedback deemed necessary. This letter contains the comments originally submitted by the editor and the reviewers followed by responses in blue italicized font for clearer reading. All line number referenced correspond to the “'Revised Manuscript with Track Changes” file.

We propose the following amended Role of Funder:

“*This work was supported by the Centers for Disease Control and Prevention/National Center for HIV, Viral Hepatitis, STD, and TB Prevention Epidemiological and Economic Modeling Agreement (NEEMA) (#5U38PS004650). Co-authors employed by the funding organization participated in the study design, interpretation, and manuscript development.”*

**Additional Editor Comments:**

Apologies for the delay. Based on the reviews we received and my own reading of the manuscript, I strongly encourage improving the exposition and clarity of the machine learning methods, particularly in response to Reviewer 2’s comments on validation procedures, performance metrics, and hyperparameter tuning. In addition, REF and its training algorithm (e.g., why not use cross-entropy?) require clearer justification.

*Thank you for your review. We have made changes to the manuscript to address the concern expressed by the reviewers. Our main point is that the validation was not conducted on the direct outputs of the Random Forest, but aggregate metrics that are more lenient of individual errors. The responses to Reviewer 2’s comments explain how the manuscript was revised for clarity.*

5. Review Comments to the Author

Reviewer #1: PONE-D-25-53291 Review

• Methods/Discussion- It would be useful for authors to clarify on what basis they made the assumption that missing data for the YRBS sexual identity question indicated non S-LGB identity, and what the implications and limitations of this assumption may be. Predicting ‘self-identity’- can the authors include some discussion on the implications (misclassification etc), and the relationship to ‘true’ sexual minority identity rates. While the rationale for the prediction of self identity in this paper is clear, given the absence of sexual orientation identity collection in the subset of YRBS participating states, and the interest of the co-author team in expanding previous prediction work in this area- it would be useful to readers and the broader literature to include some discussion of this larger context.

*Thank you for your feedback on our manuscript. We have carefully reviewed the manuscript to ensure that we stayed true to the survey questionnaire wording. Sexual identity can include multiple dimensions, including how someone labels themselves (e.g., lesbian, gay), sexual behavior, and sexual attraction. These dimensions are distinct, but no single dimension is more “true”. We have added to the discussion (lines 349-358) that future papers may employ similar methods to predict other dimensions of sexual identity such as sexual behavior. Lines 108-113 also highlight how the S-LGB percentages may not include all non-heterosexual students, which is a limitation adhering to the survey wording.*

Reviewer #2: 

Introduction, first paragraph. You state the importance of estimating percentages of youth identify as LGB for programs, including sub groups by sex, age and race/ethnicity. I agree. It’s important expand upon this statement in the first paragraph to set the stage for the rest of the manuscript. Can you give an example or two to highlight why its important to understand percentages by group and at the state level to help agencies direct healthcare resources?

*Thank you for this suggestion. We have expanded the introduction with an example between lines 69-70.*

*“*For example, state or local public health programs focused on addressing HIV risk may benefit from detailed subgroup data so outreach materials can be tailored accordingly.”

Introduction, second paragraph. “In 2021, the high school YRBS was offered in 45 states, of which 35 included a question about sexual identity.” In 2.1 Data, there is a statement that “35 states administered a high school YRBS and made the survey data publicly available”. Is it the case that the YRBS was offered in 45 states, but only 35 made the data publicly available?
*Thank you for noticing this discrepancy. We have corrected line 114 to clarify that of the 45 states that conducted the YRBS in 2021, 43 made their data publicly available through the CDC, and only 35 of those included the question pertaining to sexual identity*

Introduction, second paragraph. You state that “machine learning models have been developed to estimate the percentages of YRBS respondents who would self-identify as LGB in states …”. Farther down, you state that a “previous study demonstrated that random forest (RF) modeling produces robust estimates…” and provide a citation “[8]”. Is this the study you are referring to farther up? If so, I would present RF as the machine learning method you are referring to and insert the citation there. If there are other ML methods and citations, I would present those.
*Thank you for this comment. You are correct that both sentences refer to the same study, so we moved the citation accordingly. We also reorganized the paragraph so the sentences referring to the same study are sequential and better convey their connection.*

2.2 Estimation models by demographic subgroups, first paragraph. This paragraph refers to citation [8], mentioned in the Introduction and states that “RF obtained the best performance” among other approaches. It seems like the content from this paragraph could go in the Introduction and explain why the paper focuses on RF on the front end.

*We appreciate your suggestion. Whereas there is some repetition in justifying why our study focuses on RF, we believe the additional details are more appropriate for the “Methods” section than the Introduction.*

Table 1, Race and ethnicity. There is a category for “Native, Asian, Native Hawaiian, Other” and a “Multiracial” category with a footnote that this category is referred to as “Other” in subsequent figures. It’s confusing as to whether the other in the “Native…”’ row is different from being multiracial. Additionally, there are no N’s / percentages presented for the last three rows in the racial/ethnic category, “Native….”, “Pacific Islander, …”, and “Multiracial”.

*We apologize for the confusion. The “Other” group starts with “Non-Hispanic (…)”, so the last row of N and percentages refers to all of them. We have changed the table formatting to clarify this issue.*

2.2.1 Validation. Segmentation representations as S with superscripts for age, sex and race. (This comment applies here and throughout the paper.) I’m having trouble distinguishing between the superscripts, especially “x” and “r”. It could the way I’m viewing it and or my eyes. I’m wondering if there is a way to make this easier to view.

*This is important feedback. We have substituted the “r” to “e” for greater differentiation.*

2.2.2 Metrics. Referring to “Total Squared Error (TSE)” as a random forest performance metric for binary data, is this the Brier score, mean squared error between predicted probabilities and observed 0/1 values? Please clarify.

*Thank you for the question. No metrics are applied directly to the random forest predictions, as accurately classifying individual students is not of interest in this study. All predictions within a prediction subgroup are aggregated into an estimated percentage of S-LGB students within that subgroup, and that is the number compared to the observed LGB percentages through the TSE and ICC. We expanded the Validation segment to better highlight this detail in lines 162-165.*

“This study focuses on aggregated percentages, which provides more leeway for individual classification errors as long as the overall percentage is consistent with the observed values. This study does not aim to predict the response of individual students.”

2.2.3 Hyperparameter tuning. Is there a precedent to use ICC for hyperparameter tuning, possibly a citation? It seems like TSE and metrics focused on classification/discrimination are more commonly used. Given the hierarchical nature of the data, it’s understandable how ICC plays a role, but it’s not clear if ICC should be the primary metric versus serving as a diagnostic measure of the appropriateness of hyperparameter value selections.

*Thank you for highlighting this issue. Unfortunately, we could not find citations for the ICC being a main metric in hyper-parameter tuning. However, the metric was applied in the same way as described for concern 2.2.2. That is, the numbers being compared are not the direct output of the Random Forest classification, but the aggregate of all predictions within a given state. This aggregate is more tolerant of errors in the prediction for individual students as long as the overall percentage of the estimates approximates the real percentages of S-LGB. We were interested in estimating overall percentages across states that were consistent with the observed differences in percentages for those states. The ICC excels in measuring this consistency, so it could help us better tune the combination of hyperparameters in the RFs to the target task, rather than individual classification. We adjusted lines 173-179, 193, and 203-205 to include this justification.*

4 Discussion. “Computational complexity limited the number of options assessed in this study.” How did complexity limit options, long run times or difficulties getting the models to run? Information like that could be useful to readers to help them to understand practical limitations like if models take days to run on a desktop computer.

*Thank you for pointing out this gap. Lines 328-329 were slightly altered to clarify that this was indeed a problem of run times. Several days could be necessary to compute the estimates across all prediction subgroups. The hyperparameter tuning in particular required the repeated computation of those estimates even when only the “Aggregate” estimation approach was tested (i.e., even if a single RF is trained for each of the 35 states, the results from that tree would then be compared to other random hyper-parameter configurations. Tuning the hyperparameters for estimation approaches requiring more RFs to be trained would therefore be impractical).*
