# Supplementary material for: Indirect state-level estimation of sexual minority adolescent populations by sex, age, and race/ethnicity using random forests
Source: PLoS One. 2026 Jun 9;21(6):e0349759. doi: 10.1371/journal.pone.0349759 (PMC13249400; doi:10.1371/journal.pone.0349759)
Supplement: Peer review history

# Peer review: Decision Letter 0

**DOI:** 10.1371/journal.pone.0349759.r001

**Authors:** katsuya oi

25 Feb 2026

PONE-D-25-53291

Indirect state-level estimation of sexual minority adolescent populations by sex, age, and race/ethnicity using random forests

PLOS One

Dear Dr. Alves Maciel,

Thank you for submitting your manuscript to PLOS ONE. After careful consideration, we feel that it has merit but does not fully meet PLOS ONE’s publication criteria as it currently stands. Therefore, we invite you to submit a revised version of the manuscript that addresses the points raised during the review process.

Please submit your revised manuscript by Apr 11 2026 11:59PM. If you will need more time than this to complete your revisions, please reply to this message or contact the journal office at plosone@plos.org. When you're ready to submit your revision, log on to https://www.editorialmanager.com/pone/ and select the 'Submissions Needing Revision' folder to locate your manuscript file.

Please include the following items when submitting your revised manuscript:

- A letter that responds to each point raised by the academic editor and reviewer(s). You should upload this letter as a separate file labeled 'Response to Reviewers'.
- A marked-up copy of your manuscript that highlights changes made to the original version. You should upload this as a separate file labeled 'Revised Manuscript with Track Changes'.
- An unmarked version of your revised paper without tracked changes. You should upload this as a separate file labeled 'Manuscript'.

-->If you would like to make changes to your financial disclosure, please include your updated statement in your cover letter. Guidelines for resubmitting your figure files are available below the reviewer comments at the end of this letter.

If applicable, we recommend that you deposit your laboratory protocols in protocols.io to enhance the reproducibility of your results. Protocols.io assigns your protocol its own identifier (DOI) so that it can be cited independently in the future. For instructions see: https://journals.plos.org/plosone/s/submission-guidelines#loc-laboratory-protocols. Additionally, PLOS ONE offers an option for publishing peer-reviewed Lab Protocol articles, which describe protocols hosted on protocols.io. Read more information on sharing protocols at https://plos.org/protocols?utm_medium=editorial-email&utm_source=authorletters&utm_campaign=protocols.

We look forward to receiving your revised manuscript.

Kind regards,

katsuya oi, PhD

Academic Editor

PLOS One

**Journal Requirements:**

When submitting your revision, we need you to address these additional requirements.

1. Please ensure that your manuscript meets PLOS ONE's style requirements, including those for file naming. The PLOS ONE style templates can be found at

https://journals.plos.org/plosone/s/file?id=wjVg/PLOSOne_formatting_sample_main_body.pdf and

https://journals.plos.org/plosone/s/file?id=ba62/PLOSOne_formatting_sample_title_authors_affiliations.pdf

2. Thank you for stating the following financial disclosure:

“This work was supported by the Centers for Disease Control and Prevention/National Center for HIV, Viral Hepatitis, STD, and TB Prevention Epidemiological and Economic Modeling Agreement (NEEMA) (#5U38PS004650).”

Please state what role the funders took in the study.  If the funders had no role, please state: "The funders had no role in study design, data collection and analysis, decision to publish, or preparation of the manuscript."

If this statement is not correct you must amend it as needed.

Please include this amended Role of Funder statement in your cover letter; we will change the online submission form on your behalf.

3. Please note that funding information should not appear in any section or other areas of your manuscript. We will only publish funding information present in the Funding Statement section of the online submission form. Please remove any funding-related text from the manuscript.

4. Please upload a new copy of Figures 1, 2, 3, and Appendix Figures Reset 4-1, 4-2, 4-3, 4-4, 4-5, 4-6, 4-7, 4-8, 4-9, 5-1, 5-2, 5-3, 5-4, 5-6, and 5-7 as the detail is not clear. Please follow the link for more information:  https://journals.plos.org/plosone/s/figures

5. If the reviewer comments include a recommendation to cite specific previously published works, please review and evaluate these publications to determine whether they are relevant and should be cited. There is no requirement to cite these works unless the editor has indicated otherwise.

6. Please review your reference list to ensure that it is complete and correct. If you have cited papers that have been retracted, please include the rationale for doing so in the manuscript text, or remove these references and replace them with relevant current references. Any changes to the reference list should be mentioned in the rebuttal letter that accompanies your revised manuscript. If you need to cite a retracted article, indicate the article’s retracted status in the References list and also include a citation and full reference for the retraction notice.

**Additional Editor Comments:**

Apologies for the delay. Based on the reviews we received and my own reading of the manuscript, I strongly encourage improving the exposition and clarity of the machine learning methods, particularly in response to Reviewer 2’s comments on validation procedures, performance metrics, and hyperparameter tuning. In addition, REF and its training algorithm (e.g., why not use cross-entropy?) require clearer justification.

[Note: HTML markup is below. Please do not edit.]

Reviewers' comments:

Reviewer's Responses to Questions

**Comments to the Author**

1. Is the manuscript technically sound, and do the data support the conclusions?

The manuscript must describe a technically sound piece of scientific research with data that supports the conclusions. Experiments must have been conducted rigorously, with appropriate controls, replication, and sample sizes. The conclusions must be drawn appropriately based on the data presented.

Reviewer #1: Yes

Reviewer #2: Yes

**********

2. Has the statistical analysis been performed appropriately and rigorously?

Reviewer #1: Yes

Reviewer #2: Yes

**********

3. Have the authors made all data underlying the findings in their manuscript fully available?

The PLOS Data policy requires authors to make all data underlying the findings described in their manuscript fully available without restriction, with rare exception (please refer to the Data Availability Statement in the manuscript PDF file). The data should be provided as part of the manuscript or its supporting information, or deposited to a public repository. For example, in addition to summary statistics, the data points behind means, medians and variance measures should be available. If there are restrictions on publicly sharing data—e.g. participant privacy or use of data from a third party—those must be specified.

Reviewer #1: Yes

Reviewer #2: Yes

**********

4. Is the manuscript presented in an intelligible fashion and written in standard English?

PLOS ONE does not copyedit accepted manuscripts, so the language in submitted articles must be clear, correct, and unambiguous. Any typographical or grammatical errors should be corrected at revision, so please note any specific errors here.

Reviewer #1: Yes

Reviewer #2: Yes

**********

5. Review Comments to the Author

Please use the space provided to explain your answers to the questions above. You may also include additional comments for the author, including concerns about dual publication, research ethics, or publication ethics. (Please upload your review as an attachment if it exceeds 20,000 characters)

Reviewer #1: PONE-D-25-53291 Review

Summary:

This is a well-written manuscript that makes an important contribution to the literature on population size estimation for sexual minority populations, with applications for health disparity estimation. The use of state-level data and random forest methods to address gaps in sexual identity measure inclusion in routinely collected state level data collection is also an important contribution. Overall, the paper is well done and I include only a couple minor comments for the co-author team to address ahead of publication.

Minor comments:

• Methods- It would be useful for authors to clarify on what basis they made the assumption that missing data for the YRBS sexual identity question indicated non S-LGB identity, and what the implications and limitations of this assumption may be.

• Methods/Discussion- Predicting ‘self-identity’- can the authors include some discussion on the implications (misclassification etc), and the relationship to ‘true’ sexual minority identity rates. While the rationale for the prediction of self identity in this paper is clear, given the absence of sexual orientation identity collection in the subset of YRBS participating states, and the interest of the co-author team in expanding previous prediction work in this area- it would be useful to readers and the broader literature to include some discussion of this larger context.

Reviewer #2: This article describes the development process and results from random forests that the authors developed to impute missing self-reported lesbian, gay or bisexual (LGB) identity in Youth Risk Behavior Surveys (YRBS) among high school students across US states. In doing so, the authors present an innovative machine learning approach to impute missing values that is applicable across a wide range of applications beyond the immediate application in this article. Besides the level of innovation in the manuscript, it is well-written. Suggestions to strengthen the manuscript further follow.

Introduction, first paragraph. You state the importance of estimating percentages of youth identify as LGB for programs, including sub groups by sex, age and race/ethnicity. I agree. It’s important expand upon this statement in the first paragraph to set the stage for the rest of the manuscript. Can you give an example or two to highlight why its important to understand percentages by group and at the state level to help agencies direct healthcare resources?

Introduction, second paragraph. “In 2021, the high school YRBS was offered in 45 states, of which 35 included a question about sexual identity.” In 2.1 Data, there is a statement that “35 states administered a high school YRBS and made the survey data publicly available”. Is it the case that the YRBS was offered in 45 states, but only 35 made the data publicly available?

Introduction, second paragraph. You state that “machine learning models have been developed to estimate the percentages of YRBS respondents who would self-identify as LGB in states …”. Farther down, you state that a “previous study demonstrated that random forest (RF) modeling produces robust estimates…” and provide a citation “[8]”. Is this the study you are referring to farther up? If so, I would present RF as the machine learning method you are referring to and insert the citation there. If there are other ML methods and citations, I would present those.

2.2 Estimation models by demographic subgroups, first paragraph. This paragraph refers to citation [8], mentioned in the Introduction and states that “RF obtained the best performance” among other approaches. It seems like the content from this paragraph could go in the Introduction and explain why the paper focuses on RF on the front end.

Table 1, Race and ethnicity. There is a category for “Native, Asian, Native Hawaiian, Other” and a “Multiracial” category with a footnote that this category is referred to as “Other” in subsequent figures. It’s confusing as to whether the other in the “Native…”’ row is different from being multiracial. Additionally, there are no N’s / percentages presented for the last three rows in the racial/ethnic category, “Native….”, “Pacific Islander, …”, and “Multiracial”.

2.2.1 Validation. Segmentation representations as S with superscripts for age, sex and race. (This comment applies here and throughout the paper.) I’m having trouble distinguishing between the superscripts, especially “x” and “r”. It could the way I’m viewing it and or my eyes. I’m wondering if there is a way to make this easier to view.

2.2.2 Metrics. Referring to “Total Squared Error (TSE)” as a random forest performance metric for binary data, is this the Brier score, mean squared error between predicted probabilities and observed 0/1 values? Please clarify.

2.2.3 Hyperparameter tuning. Is there a precedent to use ICC for hyperparameter tuning, possibly a citation? It seems like TSE and metrics focused on classification/discrimination are more commonly used. Given the hierarchical nature of the data, it’s understandable how ICC plays a role, but it’s not clear if ICC should be the primary metric versus serving as a diagnostic measure of the appropriateness of hyperparameter value selections.

4 Discussion. “Computational complexity limited the number of options assessed in this study.” How did complexity limit options, long run times or difficulties getting the models to run? Information like that could be useful to readers to help them to understand practical limitations like if models take days to run on a desktop computer.

**********

6. PLOS authors have the option to publish the peer review history of their article (what does this mean?). If published, this will include your full peer review and any attached files.

If you choose “no”, your identity will remain anonymous but your review may still be made public.

**Do you want your identity to be public for this peer review?** For information about this choice, including consent withdrawal, please see our Privacy Policy.

Reviewer #1: No

Reviewer #2: **Yes:**Warren Scott Comulada

**********

[NOTE: If reviewer comments were submitted as an attachment file, they will be attached to this email and accessible via the submission site. Please log into your account, locate the manuscript record, and check for the action link "View Attachments". If this link does not appear, there are no attachment files.]

To ensure your figures meet our technical requirements, please review our figure guidelines: https://journals.plos.org/plosone/s/figures

You may also use PLOS’s free figure tool, NAAS, to help you prepare publication quality figures: https://journals.plos.org/plosone/s/figures#loc-tools-for-figure-preparation

NAAS will assess whether your figures meet our technical requirements by comparing each figure against our figure specifications.

# Author comment: Author response to Decision Letter 1

**DOI:** 10.1371/journal.pone.0349759.r002

24 Apr 2026

Reviewer 1:

Thank you for your feedback on our manuscript. We have carefully reviewed the manuscript to ensure that we stayed true to the survey questionnaire wording. Sexual identity can include multiple dimensions, including how someone labels themselves (e.g., lesbian, gay), sexual behavior, and sexual attraction. These dimensions are distinct, but no single dimension is more “true”. We have added to the discussion (lines 349-358) that future papers may employ similar methods to predict other dimensions of sexual identity such as sexual behavior. Lines 108-113 also highlight how the S-LGB percentages may not include all non-heterosexual students, which is a limitation adhering to the survey wording.

Reviewer 2:

Introduction, first paragraph. You state the importance of estimating percentages of youth identify as LGB for programs, including sub groups by sex, age and race/ethnicity. I agree. It’s important expand upon this statement in the first paragraph to set the stage for the rest of the manuscript. Can you give an example or two to highlight why its important to understand percentages by group and at the state level to help agencies direct healthcare resources?

Thank you for this suggestion. We have expanded the introduction with an example between lines 69-70.

“For example, state or local public health programs focused on addressing HIV risk may benefit from detailed subgroup data so outreach materials can be tailored accordingly.”

Introduction, second paragraph. “In 2021, the high school YRBS was offered in 45 states, of which 35 included a question about sexual identity.” In 2.1 Data, there is a statement that “35 states administered a high school YRBS and made the survey data publicly available”. Is it the case that the YRBS was offered in 45 states, but only 35 made the data publicly available?

Thank you for noticing this discrepancy. We have corrected line 114 to clarify that of the 45 states that conducted the YRBS in 2021, 43 made their data publicly available through the CDC, and only 35 of those included the question pertaining to sexual identity

Introduction, second paragraph. You state that “machine learning models have been developed to estimate the percentages of YRBS respondents who would self-identify as LGB in states …”. Farther down, you state that a “previous study demonstrated that random forest (RF) modeling produces robust estimates…” and provide a citation “[8]”. Is this the study you are referring to farther up? If so, I would present RF as the machine learning method you are referring to and insert the citation there. If there are other ML methods and citations, I would present those.

Thank you for this comment. You are correct that both sentences refer to the same study, so we moved the citation accordingly. We also reorganized the paragraph so the sentences referring to the same study are sequential and better convey their connection.

2.2 Estimation models by demographic subgroups, first paragraph. This paragraph refers to citation [8], mentioned in the Introduction and states that “RF obtained the best performance” among other approaches. It seems like the content from this paragraph could go in the Introduction and explain why the paper focuses on RF on the front end.

We appreciate your suggestion. Whereas there is some repetition in justifying why our study focuses on RF, we believe the additional details are more appropriate for the “Methods” section than the Introduction.

Table 1, Race and ethnicity. There is a category for “Native, Asian, Native Hawaiian, Other” and a “Multiracial” category with a footnote that this category is referred to as “Other” in subsequent figures. It’s confusing as to whether the other in the “Native…”’ row is different from being multiracial. Additionally, there are no N’s / percentages presented for the last three rows in the racial/ethnic category, “Native….”, “Pacific Islander, …”, and “Multiracial”.

We apologize for the confusion. The “Other” group starts with “Non-Hispanic (…)”, so the last row of N and percentages refers to all of them. We have changed the table formatting to clarify this issue.

2.2.1 Validation. Segmentation representations as S with superscripts for age, sex and race. (This comment applies here and throughout the paper.) I’m having trouble distinguishing between the superscripts, especially “x” and “r”. It could the way I’m viewing it and or my eyes. I’m wondering if there is a way to make this easier to view.

This is important feedback. We have substituted the “r” to “e” for greater differentiation.

2.2.2 Metrics. Referring to “Total Squared Error (TSE)” as a random forest performance metric for binary data, is this the Brier score, mean squared error between predicted probabilities and observed 0/1 values? Please clarify.

Thank you for the question. No metrics are applied directly to the random forest predictions, as accurately classifying individual students is not of interest in this study. All predictions within a prediction subgroup are aggregated into an estimated percentage of S-LGB students within that subgroup, and that is the number compared to the observed LGB percentages through the TSE and ICC. We expanded the Validation segment to better highlight this detail in lines 162-165.

“This study focuses on aggregated percentages, which provides more leeway for individual classification errors as long as the overall percentage is consistent with the observed values. This study does not aim to predict the response of individual students.”

2.2.3 Hyperparameter tuning. Is there a precedent to use ICC for hyperparameter tuning, possibly a citation? It seems like TSE and metrics focused on classification/discrimination are more commonly used. Given the hierarchical nature of the data, it’s understandable how ICC plays a role, but it’s not clear if ICC should be the primary metric versus serving as a diagnostic measure of the appropriateness of hyperparameter value selections.

Thank you for highlighting this issue. Unfortunately, we could not find citations for the ICC being a main metric in hyper-parameter tuning. However, the metric was applied in the same way as described for concern 2.2.2. That is, the numbers being compared are not the direct output of the Random Forest classification, but the aggregate of all predictions within a given state. This aggregate is more tolerant of errors in the prediction for individual students as long as the overall percentage of the estimates approximates the real percentages of S-LGB. We were interested in estimating overall percentages across states that were consistent with the observed differences in percentages for those states. The ICC excels in measuring this consistency, so it could help us better tune the combination of hyperparameters in the RFs to the target task, rather than individual classification. We adjusted lines 173-179, 193, and 203-205 to include this justification.

4 Discussion. “Computational complexity limited the number of options assessed in this study.” How did complexity limit options, long run times or difficulties getting the models to run? Information like that could be useful to readers to help them to understand practical limitations like if models take days to run on a desktop computer.

Thank you for pointing out this gap. Lines 328-329 were slightly altered to clarify that this was indeed a problem of run times. Several days could be necessary to compute the estimates across all prediction subgroups. The hyperparameter tuning in particular required the repeated computation of those estimates even when only the “Aggregate” estimation approach was tested (i.e., even if a single RF is trained for each of the 35 states, the results from that tree would then be compared to other random hyper-parameter configurations. Tuning the hyperparameters for estimation approaches requiring more RFs to be trained would therefore be impractical).

Attachment Submitted filename: Response to Reviewers.docx

# Editor report: Decision Letter 1

**DOI:** 10.1371/journal.pone.0349759.r003

**Authors:** katsuya oi

6 May 2026

Indirect state-level estimation of sexual minority adolescent populations by sex, age, and race/ethnicity using random forests

PONE-D-25-53291R1

Dear Dr. Alves Maciel,

We’re pleased to inform you that your manuscript has been judged scientifically suitable for publication and will be formally accepted for publication once it meets all outstanding technical requirements.

Within one week, you’ll receive an e-mail detailing the required amendments. When these have been addressed, you’ll receive a formal acceptance letter and your manuscript will be scheduled for publication.

An invoice will be generated when your article is formally accepted. Please note, if your institution has a publishing partnership with PLOS and your article meets the relevant criteria, all or part of your publication costs will be covered. Please make sure your user information is up-to-date by logging into Editorial Manager at Editorial Manager® and clicking the ‘Update My Information' link at the top of the page. For questions related to billing, please contact billing support.

If your institution or institutions have a press office, please notify them about your upcoming paper to help maximize its impact. If they’ll be preparing press materials, please inform our press team as soon as possible -- no later than 48 hours after receiving the formal acceptance. Your manuscript will remain under strict press embargo until 2 pm Eastern Time on the date of publication. For more information, please contact onepress@plos.org.

Kind regards,

katsuya oi, PhD

Academic Editor

PLOS One

Additional Editor Comments (optional):

Reviewers' comments:

# Editor report: Acceptance letter

**DOI:** 10.1371/journal.pone.0349759.r004

**Authors:** katsuya oi

PONE-D-25-53291R1

PLOS One

Dear Dr. Alves-Maciel,

I'm pleased to inform you that your manuscript has been deemed suitable for publication in PLOS One. Congratulations! Your manuscript is now being handed over to our production team.

At this stage, our production department will prepare your paper for publication. This includes ensuring the following:

* All references, tables, and figures are properly cited

* All relevant supporting information is included in the manuscript submission,

* There are no issues that prevent the paper from being properly typeset

You will receive further instructions from the production team, including instructions on how to review your proof when it is ready. Please keep in mind that we are working through a large volume of accepted articles, so please give us a few days to review your paper and let you know the next and final steps.

Lastly, if your institution or institutions have a press office, please let them know about your upcoming paper now to help maximize its impact. If they'll be preparing press materials, please inform our press team within the next 48 hours. Your manuscript will remain under strict press embargo until 2 pm Eastern Time on the date of publication. For more information, please contact onepress@plos.org.

You will receive an invoice from PLOS for your publication fee after your manuscript has reached the completed accept phase. If you receive an email requesting payment before acceptance or for any other service, this may be a phishing scheme. Learn how to identify phishing emails and protect your accounts at https://explore.plos.org/phishing.

If we can help with anything else, please email us at customercare@plos.org.

Thank you for submitting your work to PLOS ONE and supporting open access.

Kind regards,

PLOS ONE Editorial Office Staff

on behalf of

Dr. katsuya oi

Academic Editor

PLOS One
